# Combinations of Susceptibility Genes Are Associated with Higher Risk for Multiple Sclerosis and Imply Disease Course Specificity

**DOI:** 10.1371/journal.pone.0127632

**Published:** 2015-05-26

**Authors:** Denis A. Akkad, Alexandra Olischewsky, Franziska Reiner, Kerstin Hellwig, Sarika Esser, Jörg T. Epplen, Tomaz Curk, Ralf Gold, Aiden Haghikia

**Affiliations:** 1 Department of Human Genetics, Ruhr-University Bochum, Bochum, Germany; 2 Department of Neurology, St. Josef-Hospital, Ruhr-University Bochum, Bochum, Germany; 3 Faculty of Computer and Information Science, University of Ljubljana, Ljubljana, Slovenia; Friedrich-Alexander University Erlangen, GERMANY

## Abstract

Multiple sclerosis (MS) is a chronic autoimmune disease of the central nervous system that predominantly affects young adults. The genetic contributions to this multifactorial disease were underscored by a genome wide association study (GWAS) conducted by the International Multiple Sclerosis Genetic Consortium in a multinational cohort prompting the discovery of 57 non-MHC MS-associated common genetic variants. Hitherto, few of these newly reported variants have been replicated in larger independent patient cohorts. We genotyped a cohort of 1033 MS patients and 644 healthy controls with a consistent genetic background for the 57 non-MHC variants reported to be associated with MS by the first large GWAS as well as the HLA DRB1*1501 tagging SNP rs3135388. We robustly replicated three of the 57 non-MHC reported MS-associated single nucleotide polymorphisms (SNPs). In addition, our study revealed several genotype-genotype combinations with an evidently higher degree of disease association than the genotypes of the single SNPs. We further correlated well-defined clinical phenotypes, i.e. ataxia, visual impairment due to optic neuritis and paresis with single SNPs and genotype combinations, and identified several associations. The results may open new avenues for clinical implications of the MS associated genetic variants reported from large GWAS.

## Introduction

Multiple sclerosis (MS) is an immune mediated, demyelinating disease of the central nervous system and is the most common non-traumatic cause for neurologic disability in young adults in the Western world [1]. While the etiology of MS remains unknown, results of multinational and multidisciplinary projects revealed genetic and epigenetic as well as environmental influences causing MS [2]. The genetic basis of MS, like other complex multifactorial diseases, has been a matter of investigation for the last four decades. Recently, enabled by decisive progress in genetic technology, large genome-wide association studies (GWAS) have allowed for more precise investigations into this matter, coming a long way from simple linkage studies [3]. In view of many questions concerning the impact of the genetic risk in MS-etiology, two statements find consensus in the field: a) there are no rare variants with large effects following Mendelian traits that are attributable to MS, and b) there are most likely a relatively large number of common genetic variants each with small effects associated with MS which moderately add to disease risk [4]. Ensuing from an overall small risk for MS in the general population, i.e. deduced from the MS prevalence of ~0.001, it has been suggested that, even in a hypothetical scenario where all associated genetic variants have been identified, screening for them would not allow reliable prediction of MS [4].

A large international GWAS containing ~10,000 MS patients and >17,000 controls with European backgrounds from 15 countries has helped to determine genetic variants contributing to the genetic risk of MS by mapping, in an initial investigation, 57 non-MHC susceptibility loci [5], with 48 additional loci reported after further enlargement of the cohort to 29,300 MS patients and 50,794 controls [6]. Although genotyping for susceptibility loci does not seem feasible to serve as a predictive or diagnostic tool in MS, in-depth analysis of the biological role of these variants [7, 8], might prove useful both in predicting the clinical course of MS and for precision therapy.

In our current study, we genotyped 1,033 MS patients and 644 controls in an independent (from the International MS Genetics Consortium, IMSGC, and the Wellcome Trust Case Control Consortium 2, WTCCC2) cohort for the previously-reported 57 susceptibility loci [5] and the HLA DRB1*1501 tagging single nucleotide polymorphism (SNP) rs3135388. The aim of our study was to replicate previous findings, test for clinical parameters that may correlate with the genetic variations, as well as analyze whether genotype-genotype combinations and the number of risk loci present may partially explain the odds of developing MS.

## Materials and Methods

### Subjects

DNA samples were obtained via isolation from peripheral white blood cells from 1,033 unrelated German MS patients (336 males with a mean age at blood withdrawal of 42.05±11.13 years, 694 females with a mean age at blood withdrawal of 41.33±11.70 years, three samples for which patients’ sex was not confirmed). In the MS cohort, 648 patients showed relapsing remitting (RR), 229 secondary progressive (SP), 145 primary progressive (PP), and 11 clinically isolated syndrome (CIS) course according to Poser’s or McDonald’s criteria. Because collection of available DNA occurred over the last 15 years, not all patients had initially been stratified according to the McDonald criteria. Subsequent reevaluation revealed that, despite this, all CIS patients met the criteria established by McDonald et al., i.e. objective clinical evidence of one lesion [9]. SPMS was defined as continuous disability progression, i.e. motor dysfunction in the absence of or in conjunction with superimposed relapses for at least six months despite the use of immunomodulatory/immunosuppressive drugs in patients who had presented a RRMS disease course in the past. PPMS was defined as continuous disability progression without any history of past relapses. For some analyses, the MS cohort was stratified for disease progression into two subcategories: one containing only PPMS patients, the other comprised of RRMS, SPMS, and CIS patients.

DNA was also obtained from 644 age-matched healthy control subjects (380 males with a mean age of 43.28±12.05 years, 260 females with a mean age of 40.16±12.26 years, four persons with lacking sex data) residing in the Rhein-Ruhr and Hamburg areas (Germany). This current study was approved by the Ethics Committee of the Medical Faculty of the Ruhr-University Bochum, Germany (register number 4745–13). All patients and controls gave written consent for their participation.

Clinical and paraclinical data, i.e. MRI, were not available for all patients; however, correlations assessed for MRI and genotypes have been published separately [10]. For those patients whose data was available, retrospectively-acquired non-imaging clinical data obtained during regular routine examinations in our university outpatient clinic were assessed for our correlation study. The clinical data included paresis (n = 545), visual impairment due to optic neuritis (n = 545), and ataxia (n = 545) as evaluated by the functional scale scores for EDSS. These measures were correlated with either single SNPs or genotype-genotype combinations. Visual impairment due to optic neuritis included abnormal visually evoked potentials but not optical coherence tomography (OCT). The phenotypes paresis, visual impairment, and ataxia were utilized due to their quantitative nature, unlike other phenotypes consistently documented in clinical examinations. Furthermore, family history was consulted for regression analysis assessing familial clustering (n = 372), with familial history defined as an individual having any affected relatives in a direct relationship line over two generations.

### Genotyping

Investigated SNP markers were selected based on the previously published study "Genetic risk and a primary role for cell-mediated immune mechanisms in multiple sclerosis" [5] with the addition of the HLA DRB1*1501 tagging SNP rs3135388. Cohorts were genotyped for a total of 58 SNPs via TaqMan assays according to the manufacturer’s protocol (Applied Biosystems, Life Technologies) on fast PCR cycling machines (Veriti Thermal Cycler, StepOnePlus Real-Time PCR System). Genotypes were accepted for automated quality calls exceeding 98% or after manual review.

### Statistical analysis

#### Principle component analysis (PCA)

Homogeneity of the analyzed cohorts was assessed using STRUCTURE v. 2.3.4 [11]. Under the assumption of K = 3 populations, 100,000 burn-in periods, 100,000 Markov chain Monte Carlo (MCMC) replications were included after the burn-in period. Correlations, as well as independency of allele frequencies models, among the tested populations were analyzed separately. The simulations were performed 20 times for each allele frequency model, and the mean value of the proportion of membership of each pre-defined population was calculated including its corresponding standard deviation. No significant differences were observed for the two tested cohorts as indicated by the triangle plot for K = 3 populations (see Figure A in S1 File and Table A in S1 File).

#### Genotyping

Single SNP-marker testing: Allele and genotype frequencies were compared by χ^2^ testing. P-values were evaluated uncorrected as well as after Benjamini and Hochberg correction. Markers would have been excluded when Hardy-Weinberg equilibrium (HWE) yielded values less than 0.001; however, all genotyped markers, with exception of *CYP24A1* (p = 0.000583 in the control group) passed HWE-selection criteria. For completeness, the results for *CYP24A1* remain included.

Of the 58 SNPs tested, 21 (36.2%) markers share p_uncorr_-values of <0.05. After Benjamini and Hochberg correction for multiple testing, 4 (6.9%) markers pass a p_corr_-value threshold of p<0.006. Comparison of consistency for the previously-identified risk alleles reveals one deviation for the 21 (4) SNPs with p_uncorr_ <0.05 (p_corr_ <0.006) for *ZNF746* as underlined in Table 1.

**Table 1:** Single association results of the present tested 58 MS risl loci.

Gene	rs-number	p-value	risk allele present study	risk allele freq (pat / cont)	OR	95% C.I.	risk alleleIMSGC &WTCCC2	consistency of risk alleles	power
HLA*	rs3135388	2.4*10^–22^	T	0.30 / 0.15	2.414	2.014–2.892	-	-	
no gene*	rs13192841	0.00022	A	0.30 / 0.24	1.350	1.151–1.583	A	o	0.059–0.129
ZNF746*	rs354033	0.00076	A	0.26 / 0.21	1.333	1.127–1.577	G	x	0.057–0.111
TNFSF14*	rs1077667	0.00111	G	0.82 / 0.78	1.335	1.122–1.589	G	o	0.162–0.450
*MYB(AHI1)* ^ⱡ^	rs11154801	0.00623	A	0.41 / 0.36	1.222	1.058–1.411	A	o	0.099–0.265
*SCO2* ^ⱡ^	rs140522	0.00673	A	0.36 / 0.31	1.228	1.058–1.426	A	o	0.060–0.146
*SOX8* ^ⱡ^	rs2744148	0.00821	G	0.19 / 0.15	1.290	1.068–1.558	G	o	0.055–0.093
*CLECL* ^ⱡ^ *1*	rs10466829	0.00995	A	0.54 / 0.49	1.202	1.045–1.382	A	o	0.070–0.201
*CBLB* ^ⱡ^	rs2028597	0.01070	G	0.93 / 0.91	1.397	1.080–1.808	G	o	0.068–0.285
*MAPK1* ^ⱡ^	rs2283792	0.01091	C	0.54 / 0.49	1.199	1.043–1.379	C	o	0.070–0.201
*no gene* ^ⱡ^	rs669607	0.01150	C	0.53 / 0.49	1.197	1.041–1.377	C	o	0.115–0.297
*PVT1* ^ⱡ^	rs2019960	0.01471	G	0.22 / 0.18	1.244	1.044–1.483	G	o	0.054–0.094
*RPS6KB1* ^ⱡ^	rs180515	0.01748	G	0.39 / 0.35	1.192	1.031–1.378	G	o	0.073–0.176
*IRF8* ^ⱡ^	rs13333054	0.02137	A	0.23 / 0.20	1.222	1.030–1.450	A	o	0.061–0.112
*TNFRSF1A* ^ⱡ^	rs1800693	0.02419	G	0.46 / 0.42	1.176	1.021–1.354	G	o	0.091–0.234
*CXCR5* ^ⱡ^	rs630923	0.02785	C	0.86 / 0.83	1.238	1.023–1.499	C	o	0.080–0.297
*MYC* ^ⱡ^	rs4410871	0.03231	G	0.76 / 0.72	1.189	1.015–1.393	G	o	0.102–0.314
*CLEC16A(CIITA)* ^ⱡ^	rs7200786	0.03360	A	0.51 / 0.47	1.164	1.012–1.338	A	o	0.141–0.369
*CD58* ^ⱡ^	rs1335532	0.04074	A	0.90 / 0.87	1.253	1.009–1.556	A	o	0.140–0.432
*C1orf106(KIF21B)* ^ⱡ^	rs7522462	0.04080	G	0.76 / 0.73	1.182	1.007–1.387	G	o	0.088–0.283
*EVI5* ^ⱡ^	rs11810217	0.04920	A	0.27 / 0.24	1.176	1.000–1.383	A	o	0.079–0.165
*CYP27B1*	rs12368653	0.05915	A	0.51 / 0.47	1.144	0.995–1.316	A	o	0.077–0.236
*IL22RA2*	rs17066096	0.07329	G	0.29 / 0.26	1.154	0.987–1.351	G	o	0.072–0.157
*STAT3*	rs9891119	0.11601	C	0.38 / 0.35	1.124	0.972–1.300	C	o	0.067–0.155
*TMEM39A(CD80)*	rs2293370	0.14611	G	0.83 / 0.81	1.145	0.954–1.374	G	o	0.159–0.457
*no gene*	rs12466022	0.14667	C	0.75 / 0.73	1.125	0.960–1.318	C	o	0.089–0.255
*PLEK*	rs7595037	0.15508	A	0.57 / 0.54	1.107	0.962–1.274	A	o	0.083–0.235
*IL7R*	rs6897932	0.17334	G	0.75 / 0.73	1.117	0.952–1.310	G	o	0.089–0.313
*BATF*	rs2300603	0.17970	A	0.76 / 0.74	1.117	0.950–1.312	A	o	0.103–0.311
*ARL6IP4*	rs949143	0.18974	G	0.33 / 0.30	1.106	0.951–1.287	G	o	0.056–0.111
*SP140*	rs10201872	0.19638	G	0.82 / 0.80	1.123	0.942–1.340	A	x	0.110–0.376
*BACH2*	rs12212193	0.20283	A	0.54 / 0.52	1.095	0.952–1.259	G	x	0.072–0.201
*IL12B*	rs2546890	0.21215	A	0.56 / 0.54	1.094	0.950–1.259	A	o	0.083–0.232
*CD6*	rs650258	0.24279	G	0.34 / 0.36	1.091	0.943–1.263	G	o	0.079–0.175
*GALC(GPR65)*	rs2119704	0.26738	C	0.93 / 0.92	1.157	0.894–1.498	C	o	0.180–0.555
*TAGAP*	rs1738074	0.27415	G	0.63 / 0.61	1.084	0.938–1.252	G	o	0.133–0.337
*IL12A*	rs2243123	0.29910	G	0.31 / 0.29	1.084	0.931–1.263	G	o	0.059–0.124
*CD40*	rs2425752	0.30845	A	0.29 / 0.27	1.085	0.928–1.268	A	o	0.061–0.115
*IL7*	rs1520333	0.36183	G	0.28 / 0.26	1.076	0.919–1.259	G	o	0.060–0.120
*HHEX*	rs7923837	0.38237	G	0.67 / 0.65	1.068	0.922–1.238	G	o	0.089–0.256
*THEMIS*	rs802734	0.42077	A	0.72 / 0.71	1.066	0.913–1.245	A	o	0.089–0.256
*MERTK*	rs17174870	0.47272	G	0.79 / 0.78	1.064	0.899–1.259	G	o	0.087–0.271
*PTGER4*	rs4613763	0.49502	A	0.86 / 0.85	1.072	0.878–1.308	G	x	0.225–0.572
*MPV17L2(IL12RB1)*	rs874628	0.50398	A	0.25 / 0.26	1.056	0.899–1.241	A	o	0.066–0.128
*EOMES*	rs11129295	0.59737	A	0.60 / 0.61	1.039	0.901–1.199	A	o	0.100–0.302
*TYK2(ICAM3)*	rs8112449	0.60742	G	0.68 / 0.68	1.040	0.896–1.208	G	o	0.089–0.256
*RGS1*	rs1323292	0.61534	A	0.83 / 0.83	1.049	0.871–1.262	A	o	0.095–0.324
*IL2RA*	rs3118470	0.70205	G	0.67 / 0.67	1.030	0.887–1.196	G	o	0.119–0.345
*TNFRSF6B*	rs6062314	0.76479	A	0.08 / 0.08	1.041	0.802–1.350	A	o	0.053–0.066
*ZFP36L1*	rs4902647	0.79845	A	0.45 / 0.44	1.018	0.885–1.172	G	x	0.084–0.207
*NFKB1(MANBA)*	rs228614	0.80360	G	0.53 / 0.53	1.018	0.885–1.171	G	o	0.072–0.198
*CYP24A1* ^#^	rs2248359	0.81782	G	0.39 / 0.39	1.017	0.881–1.174	G	o	0.086–0.192
*CD86*	rs9282641	0.81844	G	0.08 / 0.08	1.030	0.799–1.329	G	o	0.055–0.071
*VCAM1*	rs11581062	0.83590	G	0.28 / 0.28	1.017	0.870–1.187	G	o	0.076–0.150
*MMEL1 (TNFRSF14)*	rs4648356	0.84532	A	0.29 / 0.29	1.016	0.870–1.185	C	x	0.098–0.194
*ZMIZ1*	rs1250550	0.91561	C	0.65 / 0.65	1.008	0.870–1.168	A	x	0.089–0.253
*DKKL1(CD37)*	rs2303759	0.98814	C	0.27 / 0.27	1.001	0.855–1.172	C	o	0.065–0.115
*MALT1*	rs7238078	0.98829	C	0.21 / 0.21	1.001	0.844–1.188	A	x	0.057–0.096

58 tested MS-risk loci, ranked by p-value for allele frequency comparison between MS patients and healthy controls. The left column contains the gene name used by IMSGC & WTCCC2, the second column contains the dbSNP rs-number. Column three comprises the one tailed allelic p-values calculated with one degree of freedom (df = 1). Column four represents the identified risk allele. Column five contains the odds ratios (OR) for the corresponding risk alleles. Column six depicts the 95% confidence interval (C.I.) of the corresponding OR. Column seven reflects the MS risk alleles identified and defined by the IMSGC & WTCCC2 study. Column eight shows the consistency between the MS-risk alleles as identified in the present and the IMSGC & WTCCC2 study - x = discrepancy o = consistency;

* = markers passing Benjamini and Hochberg correction for multiple testing (p_corr_-value <0.006).

^ⱡ^ markers with p_uncorr_-values <0.05.

^#^ = SNPs not passing HWE criteria. Column nine displays the power to replicate previous findings of the IMSGC & WTCCC2 study in our concise cohort for their given ORs and allele frequencies using a recessive model (most conservative) in QUANTO. Underlined corresponds to the identified inconsistency for the previously identified risk alleles with p_uncorr_ <0.05 (p_corr_ <0.006).

#### Sex effects

We stratified the cohorts with respect to sex. Allele and genotype frequencies were compared by χ^2^ testing. P-values were evaluated uncorrected as well as after Benjamini and Hochberg correction. All genotyped markers passed HWE-selection criteria (>0.001). Based on the corresponding small numbers after sex stratification, each sub-cohort was investigated for consistency of significant associations among the tested combinations: female MS patients *vs*. female controls; male MS patients *vs*. male controls; female MS patients *vs*. male MS patients and male controls *vs*. female controls.

#### Weighted genetic risk score for MS

A weighted genetic risk score (wGRS) has been previously reported using 16 established MS risk loci [12]. Here, we tested whether a higher genetic load for MS-risk factors can be identified in the MS-cohort (and sub-cohorts) in comparison to the control-cohort. Therefore calculated ORs were used as weighting factors and the amount of risk alleles as multiplicative variables (no risk allele = 0, one risk allele = 0.5, two risk alleles = 1) in order to obtain the sum above all MS-risk loci.

$$w\text{GRS}=\overset{\text{n}}{\underset{\text{i}=1}{\sum }}{\text{v}}_{\text{i}}{\text{X}}_{\text{i}}\;\text{with}\;\text{n}=58$$

**Fig 1:**
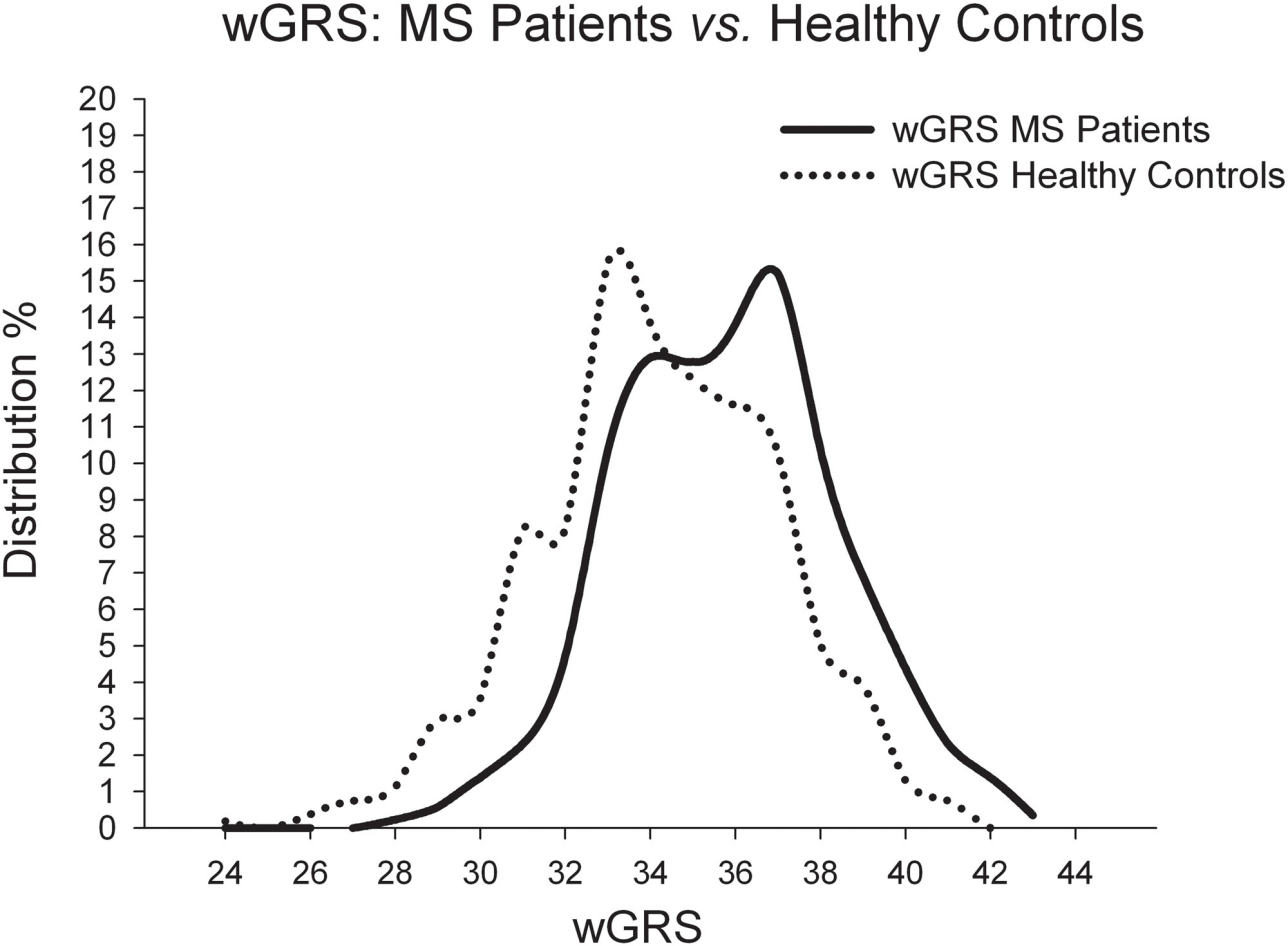
Weighted genomic risk score (wGRS) comparison. Comparison of the wGRS as determined in our MS cohort showing the percentage distribution of the wGRS in controls and MS patients. The dotted line depicts the values of the control cohort with a mean wGRS of 34.169 ± 2.784 and the continuous line depicts the MS cohort with a mean wGRS of 35.820 ± 2.621. Student’s t-test reveals significant correlation between wGRS scores and MS outcome (p = 6.5*10^–28^).

Only individuals with no missing genotypes for the investigated loci were included. We then compared the wGRS values of all groups using Student’s t-test (N_all MS_ = 868; N_controls_ = 535; N _RRMS+SPMS+CIS_ = 743; N_PPMS_ = 125). We confirmed that a significant correlation is present between wGRS scores and MS outcome (p = 6.5*10^-28^) with a mean wGRS of 34.169 ± 2.784 for controls in comparison to a wGRS of 35.820 ± 2.621 for the entire MS cohort (see Fig 1) for the tested 58 risk loci. Comparison of the MS sub-cohorts with the control-cohort yields significant differences (p = 4.2*10^–12^ for PPMS and p = 6.5*10^–25^ for RRMS+SPMS+CIS). Comparison of the MS sub-cohorts among each other revealed no significant differences with regard of wGRS and disease progression.

**Fig 2:**
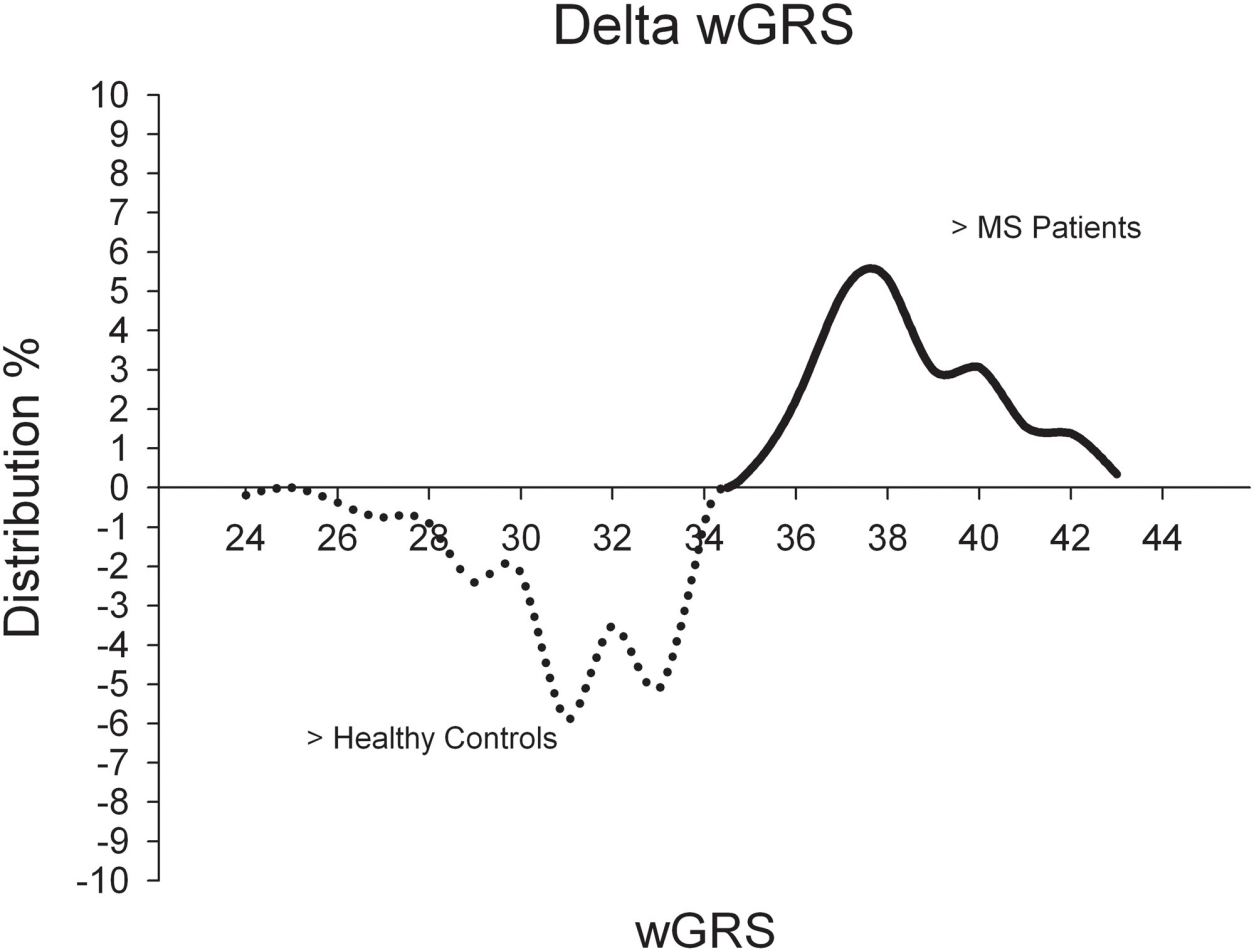
Delta wGRS. Percentage distribution of the Δ_wGRS_ = wGRS_MS_ - wGRS_controls_. The dotted line depicts a higher distribution for the corresponding wGRS in the healthy controls and the continuous line depicts a higher distribution for the corresponding wGRS in MS-patients. χ2-testing for wGRS>34.5 as risk factor, two tailed p-value 2.4*10^–16^ (OR = 2.516; 95% C.I. of 2.003–3.160).

In addition, reciprocal subtraction of the wGRS curves indicates that MS patients in general have a higher presence of wGRSs between 34.5 and 43.0 whereas controls show in general a higher frequency of wGRS between 24.0 and 34.5 (see Fig 2). χ^2^-testing for wGRS>34.5 being a risk factor reveals statistical significance for higher wGRS, with a two-tailed p-value of 2.4*10^–16^, an OR of 2.516 with a 95% C.I. of 2.003–3.160.

The wGRS was also tested for association with the age of onset and the EDSS score, for the entire as well as the sub-cohorts.

#### Regression analysis

Using regression analysis, we investigated whether certain genotype/genotype combinations led to increased MS-risk when compared to corresponding single genotype contributions. We also included testing of different inheritance models (recessive, dominant and co-dominant), and thus allele combinations, in relation to MS-manifestation by designating one of the recoded genotype combinations (see Figure C in S1 File) as risk factor and consolidating the remaining eight combinations as a group for the absence of the risk factor. χ^2^-testing was performed for all nine combinations for the corresponding models (see Figure C in S1 File), comparing all MS patients *vs*. controls, and, in addition, the MS sub-cohorts (PPMS and RR+SPMS+CIS) *vs*. the controls. The *HLA* tagging SNP was excluded from genotype-genotype analysis.

Significantly-associated genotype-genotype combinations were further processed after passing a false discovery rate (FDR) control of <0.2. In order to evaluate the contribution of given genotype-genotype combination and MS risk, we further compared the obtained combination ORs (OR_combined_) with the ORs of the underlying single genotypes (OR_single_) present in that given combination, excluding all those where no significant difference was present between OR_combined_ and the corresponding OR_single_, using following calculation:

1. $\text{Difference}\;\text{of}\;\text{thelogOdds}:\;\delta =\text{ln}\left({\text{OR}}_{\text{combined}}\right)-\text{ln}\left({\text{OR}}_{\text{single}}\right)$
2. $\text{Standard}\;\text{error}\;\text{of}\;\delta \;\text{SE}\left(\delta \right):\;\text{SE}\left(\delta \right)=\sqrt{{\text{SE}}_{\text{combined}}^{2}+{\text{SE}}_{\text{single}}^{2}}$
3. $\text{SE}\left(\text{lnOR}\right)=\sqrt{\frac{1}{{\text{risk}}_{\text{pat}}}+\frac{1}{{\text{rest}}_{\text{pat}}}+\frac{1}{{\text{risk}}_{\text{cont}}}+\frac{1}{{\text{rest}}_{\text{cont}}}}$
4. $\text{p}-\text{value}\;\text{for}\;\text{the}\;\text{ratio}\;\text{z}=\frac{\delta }{\text{SE}\left(\delta \right)}$

Genotype-genotype combinations recurring in more than one model were filtered based on best fit (p-value/OR), thus only one combination is listed for clarity. The genotype-genotype combinations passing all abovementioned criteria are listed in the Tables B-D in S1 File. In addition to the sole positive hits for each sub-cohort and model tested, we listed the results for the other cohorts and the same test category in order to estimate whether the identified genotype-genotype combination is only relevant for a MS sub cohort or the whole MS population tested.

#### Power analysis

Power analysis for the replication of previously reported risk loci [5] were conducted using QUANTO [13] under the assumption of a conservative recessive model and the reported ORs.

## Results and Discussion

In the present study, we uniformly recruited MS patients from our outpatient university clinic. Though all patients and controls originate from a confined geographic area in Germany, principle component analyses confirmed the representative nature of the data set (see Figure A in S1 File). For the phenotype correlation analyses, we included retrospective data of patients who had been monitored for clinical and paraclinical outcome measures, i.e. MRI and disease course.

Twenty-one of 58 SNPs tested in our cohort were significantly associated with MS, with the HLA DRB1*1501 tagging SNP being the strongest risk locus and 4 of the 21 passing significance threshold after correction for multiple testing (*HLA*, *no gene* rs13192841, *ZNF746*, *TNSF14*; Table 1).

Considering the power of our data set and the resulting limited probability of ~8–23% per SNP that these loci occur in independent cohorts, the confirmation of 4 (21) susceptibility loci (after and before Benjamini and Hochberg correction, respectively) underscores the robustness of the GWAS data. Important to note, the risk allele in the *ZNF746* susceptibility locus in our cohort deviated from the IMSGC&WTCC2 risk allele (see Table 1), which may represent an independent effect in our cohort. Taking into account that the majority of the individual cohorts included in the GWAS were smaller than our single center cohort (nine countries included considerably smaller numbers of MS patients [5]), our results also imply that the set of recently identified MS-associated SNPs may contain false-positive common variants and/or that population-specific susceptibility loci need to be reconsidered, e.g. by larger population specific GWAS. This line of hypothesis is supported the observation that certain loci show inverse associations within different populations, i.e. rs180515 in the French (risk allele) and the Norwegian (“protective” allele) populations [5]. Association analysis after sex stratification and subsequent Benjamini and Hochberg correction showed only one sex-specific effect: *ZFP36L1*. Both female and male MS/sex subcategories show opposing trends for genotype distribution (see Figure B in S1 File) when compared to their corresponding counterparts, possibly explaining why no significant association for *ZFP36L1* was apparent in the overall evaluation (all MS patients *vs*. all controls; see Table 1). However, this sex-specific result has to be considered with caution since the male to female ratio is 0.48:1 and 1.46:1 in the MS and control cohorts, respectively. Nevertheless, previous epidemiological studies have shown that the within-cohort sex distribution has no significant effect on the obtained results [14].

Assuming that the genetic risk of MS is determined by many common genetic variants, each with a small effect, it is feasible to conclude that individual MS patient genomes contain a higher load of disease associated SNPs than respective non-affected individuals in the same population [4]. This assumption has been translated into a predictive value for MS detection in a study which utilized 16 known susceptibility loci [12]. It has also previously been suggested that the accumulation of disease-associated SNPs is likely to differ in individual MS patients in multi-case families from sporadic cases, with familial cases having a higher load of associated SNPs [15]. In order to evaluate whether the SNP load in our MS cohort revealed differences from the controls, we assessed the wGRS, which considers the number and relative contribution of the risk alleles, as previously reported [12] based on the 58 susceptibility loci genotyped in our MS and control cohort. Indeed, the SNP load in MS patients [mean wGRS = 35.820 ± 2.621; p = 6.5*10^–28^] was significantly higher than in the control group [mean wGRS = 34.169 ± 2.784], within the whole cohort (not distinguishing between familial and sporadic MS). Moreover, reciprocal subtraction of the wGRS curves indicates that MS patients in general have a higher wGRS (between 34.5 and 43.0), whereas controls show a frequency of wGRS values between 24.0 and 34.5. Estimating the probability to develop MS based on a wGRS>34.5, we were able to confirm that an increased load of risk SNP alleles clearly elevates the risk for MS (OR = 2.516, 95% C.I. of 2.003–3.160, Figs 1 and 2). Correlation of the wGRS with age of onset and EDSS score, for the entire as well as the corresponding sub-cohorts, revealed no significant associations. As discussed in prior studies, higher wGRS scores are of very limited value in MS. Yet, these findings underscore the concept of cumulative genetic risks in MS through the additive effect of tiny contributions by common genetic variants [4].

**Fig 3:**
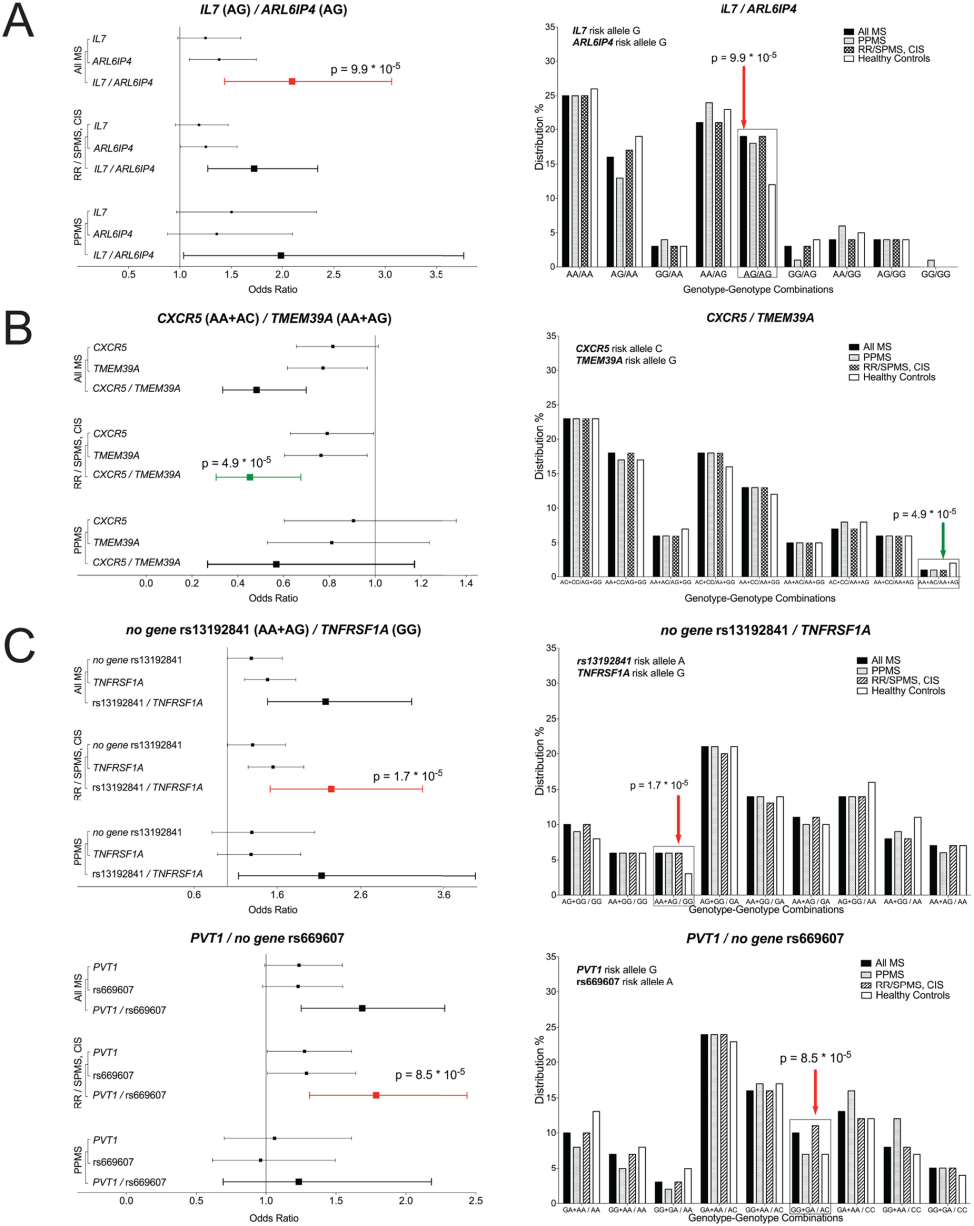
Forest Plots and genotype-genotype distributions for significantly associated combinations. Left-hand side: Forest plots showing the odds ratios (OR; incl. 95% C.I.) for the significantly associated genotype-genotype combinations, including the contributing single genotype ORs. Lead genotype-genotype combinations have been listed and colored for ORs with a p-value <1*10^–4^, red = OR>1, green = OR<1. ORs have been listed for all tested cohorts/disease courses (All MS; RRMS, SPMS and CIS; PPMS) in order to distinguish disease course specificity. The contributing genotype is stated in brackets behind the corresponding heading gene name. On the right handed side, the percentage distribution of each genotype-genotype combination in the given cohort is presented as a bar chart. The significantly associated genotype-genotype combination from the left handed graphic is highlighted via frame and colored arrow; red = OR>1, green = OR<1. A, B, C: correspond to significantly associated genotype-genotype combinations obtained from models A, B and C.

We next evaluated whether single SNP-SNP genotype combinations within the 57 non-HLA susceptibility loci, including different dominance models for any two risk-loci, exerted a higher risk for MS than the respective single risk genotypes. Risk association was analyzed for the MS patients vs. controls and for different clinical subgroups stratification (RRMS and SPMS v. PPMS). To test our hypothesis, we evaluated a total of 14,364 possible genotype combinations for each group and inheritance model (all MS; RR/SPMS and CIS were considered one group considering the clinical course of MS; PPMS; controls) by binary logistic regression analyses. To reduce statistic artifacts to a minimum, we rigorously discarded all combinations with a higher FDR of > 0.2. Our analyses revealed in total 37 genotype-genotype combinations possibly contributing greater to MS pathogenesis than their underlying single genotypes, four of which show p-values <1*10^–4^ that were significantly associated either with ORs>1 or ORs<1 from either the entire MS, or RR/SPMS as depicted for the different scenarios in Fig 3. All four genotype-genotype combinations revealed higher or respectively lower ORs than the single genotypes and, of special relevance, seven loci that were not significantly associated after correction with MS in our cohort were represented in these extended genotype-genotype combinations (*IL7*, *ARL6IP4*, *CXCR5*, *TMEM39A(CD80)*, *TNFRSF1A*, *no gene* rs669607, *PVT1;* Table 1 and Fig 3). Despite the size of this single center cohort, we could not overcome the limitation of statistical power with respect to the SNP-SNP combination analysis after sex stratification. Therefore, we cannot exclude the possibility that sex, and in this present cohort also sex composition, may reveal further SNP-SNP genotype combinations which should be addressed in future larger GWAS.

**Fig 4:**
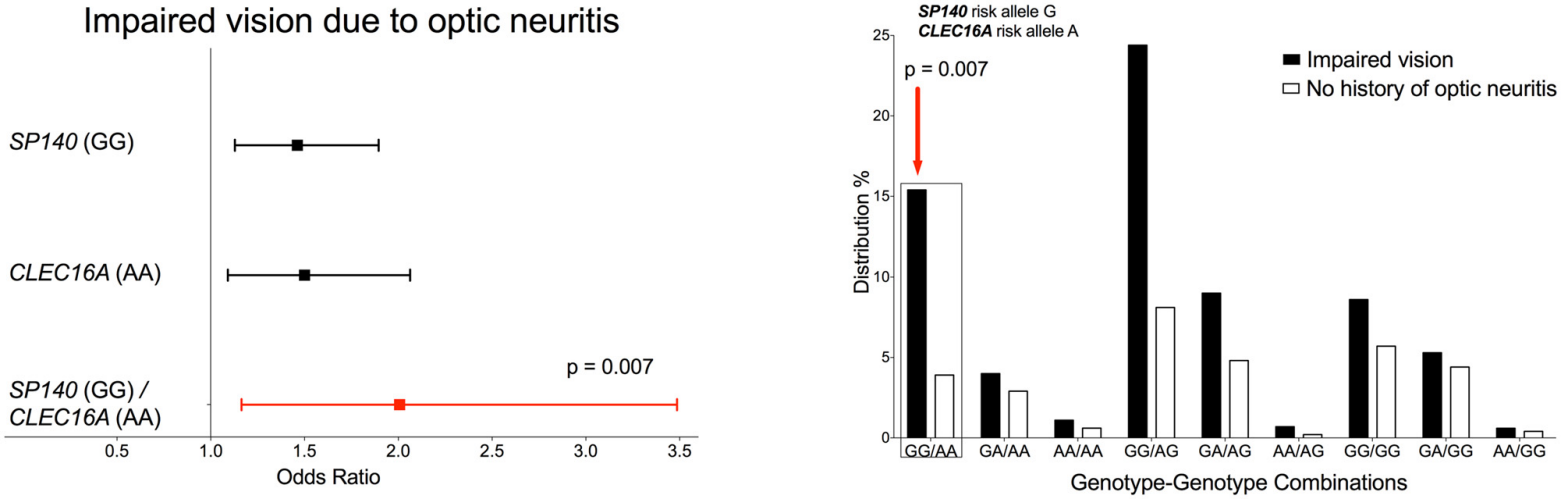
Forest Plot and genotype-genotype distributions for the significantly associated combination with visual impairment. Left-hand side: Forest plot showing the odds ratio (OR; incl. 95% C.I.) for the significantly with visual impairment associated genotype-genotype combination, including the contributing single genotype ORs. The lead genotype-genotype combination has been listed and colored in red for OR>1. The contributing genotype is stated in brackets behind the corresponding heading gene name. On the right handed side the percentage distribution of each genotype-genotype combination in the given cohort is presented as a bar chart. The significantly associated genotype-genotype combination from the left handed graphic is highlighted via frame and colored arrow; red = OR>1.

In our extended study, we correlated well-defined MS clinical phenotypes, i.e. paresis, visual impairment due to optic neuritis, and ataxia with MS associated SNPs. Here, we saw several single genetic variations that revealed significant correlation with the candidate clinical phenotypes assessed in our study (see Table E in S1 File). Interestingly, we also found an association of identified genotype-genotype combinations with these clinical phenotypes which seem to outweigh the single SNP effect as shown for the prevalence of visual impairment (as shown in Fig 4 for visual impairment and Table F in S1 File). However, due to limited available data, the clinical correlation data need to be interpreted with caution with respect to statistical robustness. At most, the present study may indicate the possibility how GWAS can be applied to the clinical context.

Our replication data of associated SNPs identified by the latest GWAS suggest that larger, population-specific GWASs are needed to identify all potentially population-specific relevant MS-associated genetic variants. While this paradigm opposes the notion that MS shares the same causal pathogenetic features among different populations, it is important to remember that all individual MS-risk alleles confer a small contribution to the greater disease course. The interaction of (risk) genes among each other [16], as shown in other autoimmune disorders such as lupus erythematosus [17], and with environmental risk factors, as recently demonstrated for esophageal malignancies [18] and previously for MS [19], will most likely differ within populations with diverse genetic backgrounds and lead to innumerable complex combinations that predispose for MS. For instance, it seems rather unlikely that Vitamin D deficiency, a suspected environmental risk factor for MS, has the same risk effect among populations situated in distant- vs. near-equatorial locations. Based on recently-identified risk loci, our data further imply that genotype-genotype combinations of MS associated SNPs reveal higher risks for MS than corresponding single SNPs (Fig 3 and Tables B-D in S1 File), bearing in mind that the analyzed SNPs may just tag the MS-relevant variations. This observation is consistent with previously proposed gene-protein pathway analyses based on mathematical models [20]. These results require confirmation in large independent replication studies, and, importantly, they require functional correlates before assumptions about pathogenetic gene-gene combination effects can be truly made. In view of additional MS associated risk loci expected to be reported soon, it is vital to elaborate further on how these loci contribute to disease.

## Supporting Information

S1 File Figure A. Principle component analysis (PCA). Figure B. *ZFP36L1* genotype distributions after gender stratification. Figure C. Recoded genotype combinations. Table A. PCA proportion. Table B. p-value and ORs for genotype/genotype combinations for model A). Table C. p-value and ORs for genotype/genotype combinations for model B). Table D. p-value and ORs for genotype/genotype combinations for model C). Table E. p-value and ORs for allele correlation with clinical parameters. Table F. p-value and ORs for genotype/genotype combinations according to Model A) correlated for clinical parameters.
(DOCX)
