# Supplementary material for: Combinations of Susceptibility Genes Are Associated with Higher Risk for Multiple Sclerosis and Imply Disease Course Specificity
Source: PLoS One. 2015 May 26;10(5):e0127632. doi: 10.1371/journal.pone.0127632 (PMC4444291; doi:10.1371/journal.pone.0127632)
Supplement: S1 File — Figure A. Principle component analysis (PCA). Figure B. ZFP36L1 genotype distributions after gender stratification. Figure C. Recoded genotype combinations. Table A. PCA proportion. Table B. p-value and ORs for genotype/genotype combinations for model A). Table C. p-value and ORs for genotype/genotype combinations for model B). Table D. p-value and ORs for genotype/genotype combinations for model C). Table E. p-value and ORs for allele correlation with clinical parameters. Table F. p-value and ORs for genotype/genotype combinations according to Model A) correlated for clinical parameters. (DOCX) [file pone.0127632.s001.docx]

**Supplementary methods and results**

**Susceptibility genes are associated with higher risk for Multiple Sclerosis in certain combinations and imply disease course specificity**

**Denis A. Akkad^1^, Alexandra Olischewsky^2^, Franziska Reiner^2^, Kerstin Hellwig^2^, Sarika Esser^1^, Jörg T. Epplen^1^, Tomaz Curk^3^, Ralf Gold^2^, Aiden Haghikia^2^**

^1^ Department of Human Genetics, Ruhr-University Bochum, Germany

^3^ Department of Neurology, St. Josef-Hospital, Ruhr-University Bochum, Germany

^2^ Faculty of Computer and Information Science, University of Ljubljana, Slovenia

Addresses for correspondence:

[amer.akkad@ruhr-uni-bochum.de](mailto:amer.akkad@ruhr-uni-bochum.de)

aiden.haghikia@ruhr-uni-bochum.de


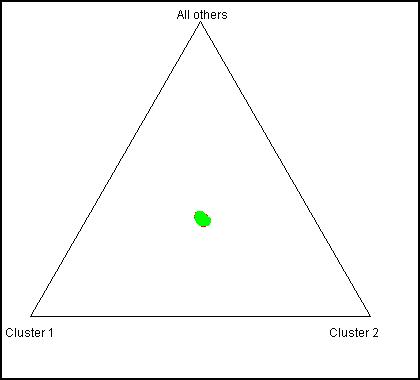


**Figure A in S1. Principle component analysis (PCA).** Exemplary triangle plot for K=3 populations using PCA-analysis; red dots represent control individuals green dots represent MS patients. The bottom left corner is assigned to population one, the bottom right corner to population two and the top corner represents all other populations in our case population three.

| allele frequency model | population | inferred clusters | | | number of individuals |
| --- | --- | --- | --- | --- | --- |
|  |  | 1 | 2 | 3 |  |
| correlated | Contr | 0.3335±0.0005 | 0.3333±0.0004 | 0.3330±0.0003 | 644 |
|  | MS | 0.3332±0.0004 | 0.3330±0.0000 | 0.3331±0.0002 | 1033 |
| independent | Contr | 0.3334±0.0005 | 0.3333±0.0005 | 0.3332±0.0004 | 644 |
|  | MS | 0.3332±0.0004 | 0.3333±0.0004 | 0.3331±0.0003 | 1033 |

**Table A in S1.** **PCA proportion.** Proportion of membership of each pre-defined population in each of the three clusters as defined by STRUCTURE v. 2.3.4. The simulation has been performed 20 times for each allele frequency model leading to corresponding mean values incl. standard deviations for the inferred clusters. Contr: controls/healthy blood donors; MS: multiple sclerosis patients.


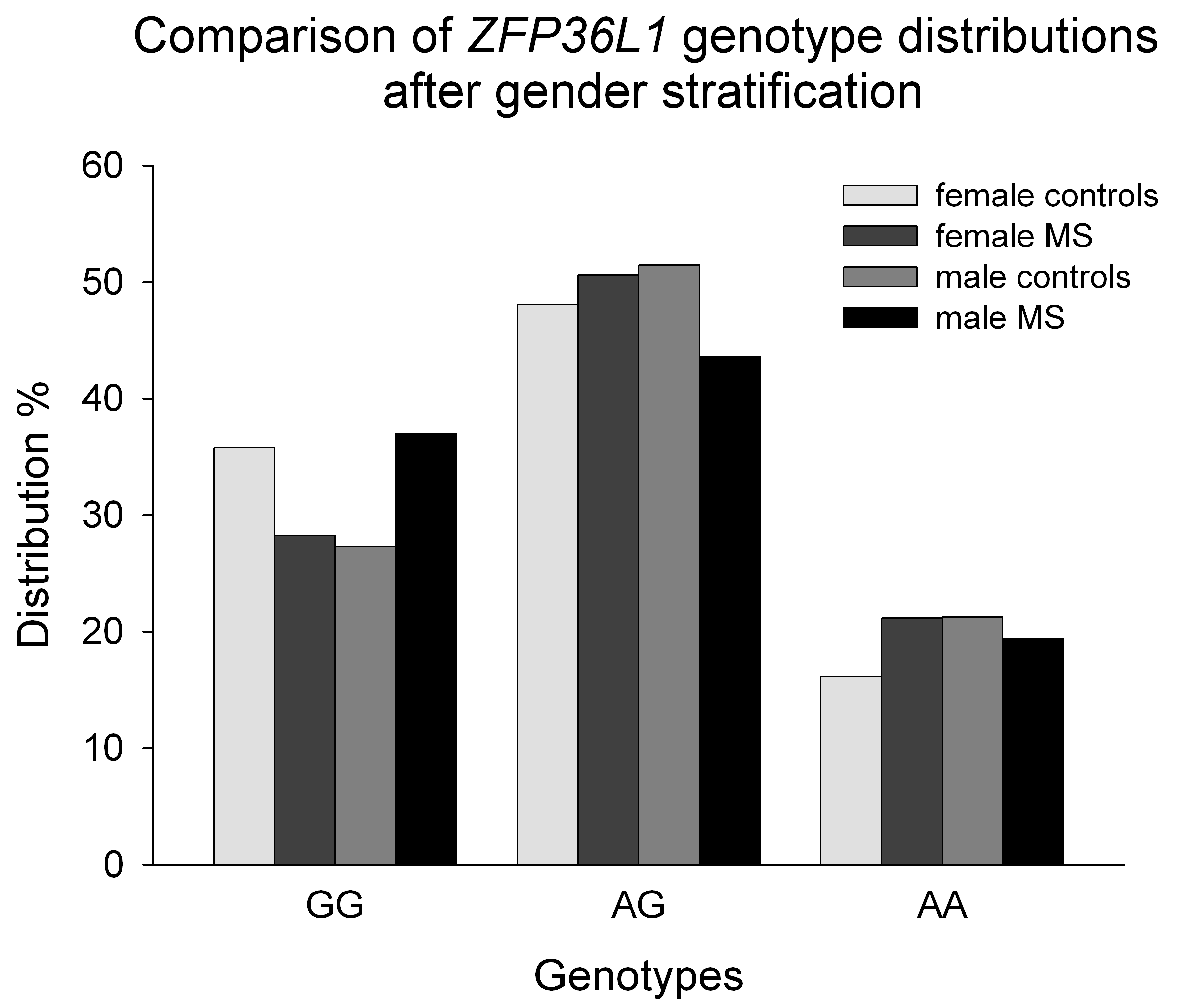


**Figure B in S1. *ZFP36L1* genotype distributions after gender stratification.** Comparison for the allelic distribution for female MS patients *vs.* female controls yields a p_uncorr_-value of 0.01446 and a corresponding OR of 1.291, a 95% C.I. of 1.052-1.584 with allele 2 being the risk allele. Comparison for the allelic distribution for male MS patients *vs.* male controls yields a p_uncorr_-value of 0.02906 and a corresponding OR of 1.263, a 95% C.I. of 1.024-1.559 with allele 1 representing the risk allele.


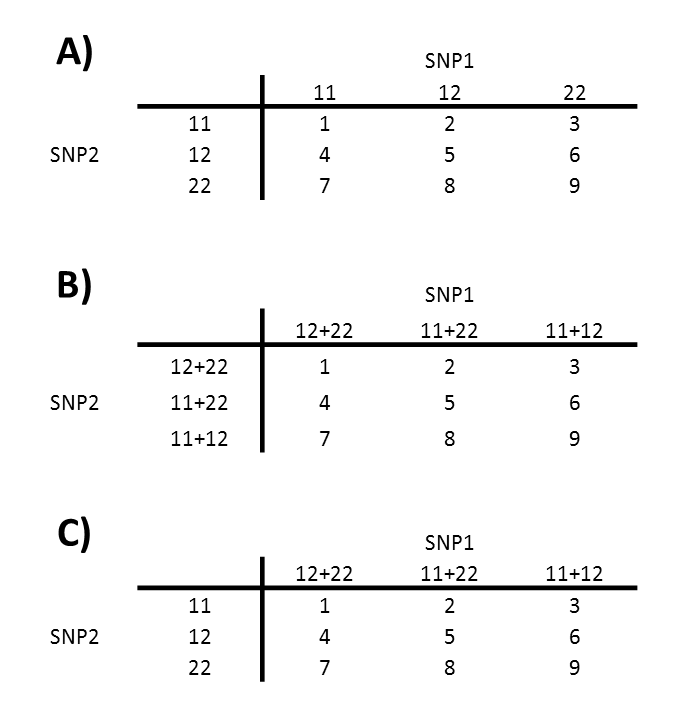


**Figure C in S1.** **Recoded genotype combinations.** SNP1 and SNP2 risk combination possibilities for regression analysis using χ^2^-testing with regard to different interaction modes. A) depicts the possible contribution of a distinct single genotype (from left to right: autosomal recessive 1; co-dominant 12; autosomal recessive 2) at SNP1 with the possible contribution of a distinct single genotype (from top to bottom: autosomal recessive 1; co-dominant 12; autosomal recessive 2) at SNP2. B) reflects the contribution of dominance effects for distinct genotypes (from left to right: allele 2 being dominant over allele 1; no co-dominant 12 genotype present; allele 1 being dominant over allele 2) at SNP1 with the possible contribution of distinct genotypes (from top to bottom: allele 2 being dominant over allele 1; no co-dominant genotype 12 present; allele 1 being dominant over allele 2) at SNP2. C) reflects the contribution of dominance effects for distinct genotypes (from left to right: allele 2 being dominant over allele 1; no co-dominant genotype 12 present; allele 1 being dominant over allele 2) at SNP1 with the possible contribution of a distinct single genotype (from top to bottom: autosomal recessive 1; co-dominant 12; autosomal recessive 2) at SNP2

| Model A) |  |  |  |  |  |  |  |  |  |  |  |  |  |  |  |  |  |  |  |
| --- | --- | --- | --- | --- | --- | --- | --- | --- | --- | --- | --- | --- | --- | --- | --- | --- | --- | --- | --- |
| all MS vs. controls | | |  |  |  |  | SNP1-SNP2 combination | | | | | SNP1 | | | | SNP2 | | | |
| SNP1 | SNP2 | Gene 1 | Gene 2 | Coding | SNP1 | SNP2 | p val | OR | 95% C.I. | Pat | Contr | OR | 95% C.I. | Pat | Contr | OR | 95% C.I. | Pat | Contr |
| rs1520333 | rs949143 | IL7 | ARL6IP4 | 5 | AG | AG | 9.97*10^-05 C)^ | 1.748 | 1.302-2.350 | 198/827 | 76/555 | 1.248 | 1.013-1.539 | 418/608 | 228/414 | 1.190 | 0.968-1.462 | 456/572 | 254/379 |
| rs669607 | rs12368653 | no gene | CYP27B1 | 7 | AA | GG | 1.09*10^-04 B)^ | 0.481 | 0.327-0.706 | 56/971 | 68/567 | 0.699 | 0.552-0.885 | 215/814 | 175/463 | 0.837 | 0.666-1.052 | 258/769 | 182/454 |
| rs10466829 | rs13333054 | CLECL1 | IRF8 | 3 | GG | GG | 1.24*10^-04 C)^ | 0.578 | 0.434-0.769 | 119/904 | 118/518 | 0.760 | 0.599-0.964 | 219/808 | 168/471 | 0.779 | 0.632-0.961 | 601/424 | 411/226 |
| rs10466829 | rs2546890 | CLECL1 | IL12B | 9 | GG | GG | 2.41*10^-04 C)^ | 0.428 | 0.265-0.688 | 34/991 | 47/586 | 0.760 | 0.599-0.964 | 219/808 | 168/471 | 0.970 | 0.754-1.248 | 209/818 | 132/501 |
| rs7595037 | rs669607 | PLEK | no gene | 1 | GG | AA | 3.23*10^-04 C)^ | 0.454 | 0.289-0.712 | 39/987 | 51/586 | 0.841 | 0.657-1.077 | 205/821 | 146/492 | 0.699 | 0.552-0.885 | 215/814 | 175/463 |
| rs140522 | rs13333054 | SCO2 | IRF8 | 5 | AG | AG | 4.10*10^-04 C)^ | 1.697 | 1.251-2.304 | 180/844 | 71/565 | 1.191 | 0.972-1.461 | 486/540 | 275/364 | 1.251 | 1.007-1.554 | 368/657 | 197/440 |
| rs13192841 | rs11154801 | no gene | MYB(AHI1) | 2 | AG | AA | 4.13*10^-04 C)^ | 2.381 | 1.403-4.077 | 73/949 | 20/619 | 1.397 | 1.131-1.724 | 428/596 | 217/422 | 1.259 | 0.939-1.69 | 164/862 | 84/556 |
| rs669607 | rs2744148 | no gene | SOX8 | 6 | CC | AG | 8.63*10^-04 D)^ | 2.040 | 1.302-3.213 | 91/935 | 29/608 | 1.148 | 0.911-1.448 | 284/745 | 159/479 | 1.221 | 0.974-1.53 | 317/709 | 171/467 |
| rs140522 | rs11154801 | SCO2 | MYB(AHI1) | 2 | AG | AA | 9.45*10^-04 D)^ | 2.178 | 1.322-3.611 | 77/947 | 23/616 | 1.191 | 0.972-1.461 | 486/540 | 275/364 | 1.259 | 0.939-1.69 | 164/862 | 84/556 |
| rs7200786 | rs9282641 | CLEC16A(CIITA) | CD86 | 4 | AA | AG | 1.13*10^-03 D)^ | 2.948 | 1.424-6.272 | 46/972 | 10/623 | 1.173 | 0.924-1.49 | 264/765 | 145/493 | 1.068 | 0.801-1.426 | 156/862 | 92/543 |
| rs10201872 | rs7200786 | SP140 | CLEC16A(CIITA) | 1 | *GG* | AA | 1.14*10^-03 D)^ | 1.601 | 1.191-2.154 | 186/842 | 77/558 | 1.134 | 0.917-1.402 | 682/346 | 405/233 | 1.173 | 0.924-1.49 | 264/765 | 145/493 |
| rs11154801 | rs2300603 | MYB(AHI1) | BATF | 3 | CC | GG | 1.28*10^-03 D)^ | 0.340 | 0.167-0.687 | 14/1009 | 25/613 | 0.752 | 0.61-0.927 | 350/676 | 261/379 | 0.800 | 0.521-1.231 | 56/970 | 43/596 |
| rs10466829 | rs7200786 | CLECL1 | CLEC16A(CIITA) | 9 | GG | GG | 1.34*10^-03 D)^ | 0.506 | 0.331-0.775 | 46/981 | 54/583 | 0.760 | 0.599-0.964 | 219/808 | 168/471 | 0.788 | 0.626-0.991 | 249/780 | 184/454 |
| rs180515 | rs228614 | RPS6KB1 | NFKB1(MANBA) | 9 | GG | GG | 1.43*10^-03 D)^ | 2.523 | 1.349-4.792 | 55/967 | 14/621 | 1.317 | 0.974-1.781 | 158/866 | 78/563 | 0.970 | 0.771-1.222 | 269/757 | 171/467 |
| rs7522462 | rs13192841 | C1orf106(KIF21B) | no gene | 3 | GG | AA | 1.76*10^-03 D)^ | 2.458 | 1.34-4.574 | 57/960 | 15/621 | 1.202 | 0.981-1.475 | 598/424 | 346/295 | 1.303 | 0.885-1.921 | 92/932 | 45/594 |
| rs11129295 | rs12368653 | EOMES | CYP27B1 | 7 | GG | GG | 1.99*10^-03 D)^ | 0.589 | 0.419-0.827 | 80/939 | 80/553 | 0.913 | 0.739-1.126 | 363/658 | 240/397 | 0.837 | 0.666-1.052 | 258/769 | 182/454 |
| rs10466829 | rs10201872 | CLECL1 | SP140 | 6 | GG | *AG* | 2.72*10^-03 E)^ | 0.566 | 0.385-0.831 | 60/966 | 63/574 | 0.760 | 0.599-0.964 | 219/808 | 168/471 | 0.913 | 0.735-1.136 | 315/713 | 208/430 |
|  |  |  |  |  |  |  |  |  |  |  |  |  |  |  |  |  |  |  |  |
| RR/SPMS+CIS vs. controls | | |  |  |  |  | SNP1-SNP2 combination | | | | | SNP1 | | | | SNP2 | | | |
| SNP1 | SNP2 | Gene 1 | Gene 2 | Coding | SNP1 | SNP2 | p val | OR | 95% C.I. | Pat | Contr | OR | 95% C.I. | Pat | Contr | OR | 95% C.I. | Pat | Contr |
| rs1520333 | rs949143 | IL7 | ARL6IP4 | 5 | AG | AG | 2.46*10^-04 D)^ | 1.726 | 1.272-2.344 | 160/677 | 76/555 | 1.252 | 1.007-1.557 | 342/496 | 228/414 | 1.186 | 0.957-1.47 | 372/468 | 254/379 |
| rs669607 | rs12368653 | no gene | CYP27B1 | 7 | AA | GG | 3.79*10^-04 E)^ | 0.494 | 0.330-0.740 | 47/793 | 68/567 | 0.720 | 0.563-0.922 | 180/661 | 175/463 | 0.816 | 0.642-1.037 | 207/633 | 182/454 |
| rs10466829 | rs13333054 | CLECL1 | IRF8 | 3 | GG | GG | 1.29*10^-04 E)^ | 0.563 | 0.416-0.762 | 95/741 | 118/518 | 0.766 | 0.597-0.982 | 180/659 | 168/471 | 0.770 | 0.619-0.958 | 489/349 | 411/226 |
| rs10466829 | rs2546890 | CLECL1 | IL12B | 9 | GG | GG | 8.07*10^-04 D)^ | 0.447 | 0.271-0.737 | 29/808 | 47/586 | 0.766 | 0.597-0.982 | 180/659 | 168/471 | 0.979 | 0.753-1.273 | 172/667 | 132/501 |
| rs7595037 | rs669607 | PLEK | no gene | 1 | GG | AA | 3.00*10^-04 C)^ | 0.427 | 0.262-0.694 | 30/808 | 51/586 | 0.814 | 0.628-1.055 | 163/675 | 146/492 | 0.720 | 0.563-0.922 | 180/661 | 175/463 |
| rs140522 | rs13333054 | SCO2 | IRF8 | 5 | AG | AG | 8.13*10^-04 E)^ | 1.681 | 1.227-2.307 | 146/691 | 71/565 | 1.144 | 0.925-1.415 | 389/450 | 275/364 | 1.265 | 1.01-1.585 | 303/535 | 197/440 |
| rs13192841 | rs11154801 | no gene | MYB(AHI1) | 2 | AG | AA | 3.34*10^-04 C)^ | 2.479 | 1.444-4.292 | 62/774 | 20/619 | 1.446 | 1.162-1.801 | 357/480 | 217/422 | 1.292 | 0.953-1.751 | 137/702 | 84/556 |
| rs669607 | rs2744148 | no gene | SOX8 | 6 | CC | AG | 2.43*10^-03 E)^ | 1.971 | 1.238-3.149 | 72/766 | 29/608 | 1.061 | 0.832-1.353 | 219/622 | 159/479 | 1.201 | 0.949-1.52 | 256/582 | 171/467 |
| rs140522 | rs11154801 | SCO2 | MYB(AHI1) | 2 | AG | AA | 5.72*10^-04 D)^ | 2.290 | 1.375-3.837 | 66/772 | 23/616 | 1.144 | 0.925-1.415 | 389/450 | 275/364 | 1.292 | 0.953-1.751 | 137/702 | 84/556 |
| rs7200786 | rs9282641 | CLEC16A(CIITA) | CD86 | 4 | AA | AG | 1.59*10^-03 E)^ | 2.978 | 1.416-6.427 | 38/795 | 10/623 | 1.197 | 0.934-1.534 | 219/622 | 145/493 | 1.101 | 0.817-1.486 | 131/702 | 92/543 |
| rs10201872 | rs7200786 | SP140 | CLEC16A(CIITA) | 1 | *GG* | AA | 1.13*10^-03 E)^ | 1.624 | 1.197-2.207 | 154/687 | 77/558 | 1.140 | 0.914-1.423 | 559/282 | 405/233 | 1.197 | 0.934-1.534 | 219/622 | 145/493 |
| rs11154801 | rs2300603 | MYB(AHI1) | BATF | 3 | CC | GG | 1.82*10^-03 E)^ | 0.327 | 0.150-0.700 | 11/825 | 25/613 | 0.751 | 0.604-0.934 | 286/553 | 261/379 | 0.824 | 0.526-1.289 | 47/791 | 43/596 |
| rs10466829 | rs7200786 | CLECL1 | CLEC16A(CIITA) | 9 | GG | GG | 2.24*10^-03 E)^ | 0.512 | 0.326-0.802 | 38/801 | 54/583 | 0.766 | 0.597-0.982 | 180/659 | 168/471 | 0.780 | 0.614-0.992 | 202/639 | 184/454 |
| rs180515 | rs228614 | RPS6KB1 | NFKB1(MANBA) | 9 | GG | GG | 3.90*10^-03 E)^ | 2.408 | 1.263-4.659 | 43/792 | 14/621 | 1.329 | 0.972-1.818 | 130/706 | 78/563 | 0.971 | 0.764-1.234 | 220/619 | 171/467 |
| rs7522462 | rs13192841 | C1orf106(KIF21B) | no gene | 3 | GG | AA | 4.62*10^-03 E)^ | 2.315 | 1.236-4.391 | 44/787 | 15/621 | 1.147 | 0.927-1.419 | 479/356 | 346/295 | 1.337 | 0.897-1.997 | 77/760 | 45/594 |
| rs11129295 | rs12368653 | EOMES | CYP27B1 | 7 | GG | GG | 6.13*10^-04 D)^ | 0.537 | 0.372-0.775 | 60/772 | 80/553 | 0.893 | 0.716-1.113 | 292/541 | 240/397 | 0.816 | 0.642-1.037 | 207/633 | 182/454 |
| rs10466829 | rs10201872 | CLECL1 | SP140 | 6 | GG | *AG* | 1.17*10^-03 D)^ | 0.516 | 0.341-0.782 | 45/794 | 63/574 | 0.766 | 0.597-0.982 | 180/659 | 168/471 | 0.884 | 0.704-1.111 | 252/589 | 208/430 |
|  |  |  |  |  |  |  |  |  |  |  |  |  |  |  |  |  |  |  |  |
| PPMS vs. controls | | |  |  |  |  | SNP1-SNP2 combination | | | | | SNP1 | | | | SNP2 | | | |
| SNP1 | SNP2 | Gene 1 | Gene 2 | Coding | SNP1 | SNP2 | p val | OR | 95% C.I. | Pat | Contr | OR | 95% C.I. | Pat | Contr | OR | 95% C.I. | Pat | Contr |
| rs1520333 | rs949143 | IL7 | ARL6IP4 | 5 | AG | AG | 7.46*10^-02 E)^ | 1.609 | 0.959-2.688 | 26/118 | 76/555 | 1.058 | 0.714-1.565 | 53/91 | 228/414 | 1.161 | 0.792-1.7 | 63/81 | 254/379 |
| rs669607 | rs12368653 | no gene | CYP27B1 | 7 | AA | GG | 1.71*10^-02 E)^ | 0.363 | 0.139-0.890 | 6/138 | 68/567 | 0.529 | 0.321-0.867 | 24/120 | 175/463 | 1.027 | 0.676-1.558 | 42/102 | 182/454 |
| rs10466829 | rs13333054 | CLECL1 | IRF8 | 3 | GG | GG | 2.26*10^-01 E)^ | 0.708 | 0.410-1.213 | 20/124 | 118/518 | 0.801 | 0.509-1.257 | 32/112 | 168/471 | 0.864 | 0.586-1.276 | 88/56 | 411/226 |
| rs10466829 | rs2546890 | CLECL1 | IL12B | 9 | GG | GG | 1.43*10^-02 E)^ | 0.265 | 0.065-0.902 | 3/141 | 47/586 | 0.801 | 0.509-1.257 | 32/112 | 168/471 | 1.041 | 0.653-1.653 | 31/113 | 132/501 |
| rs7595037 | rs669607 | PLEK | no gene | 1 | GG | AA | 7.17*10^-02 E)^ | 0.413 | 0.142-1.105 | 5/139 | 51/586 | 0.963 | 0.609-1.517 | 32/112 | 146/492 | 0.529 | 0.321-0.867 | 24/120 | 175/463 |
| rs140522 | rs13333054 | SCO2 | IRF8 | 5 | AG | AG | 4.88*10^-02 E)^ | 1.672 | 0.986-2.821 | 25/119 | 71/565 | 1.439 | 0.985-2.101 | 75/69 | 275/364 | 1.152 | 0.771-1.72 | 49/95 | 197/440 |
| rs13192841 | rs11154801 | no gene | MYB(AHI1) | 2 | AG | AA | 2.09*10^-01 E)^ | 1.821 | 0.720-4.471 | 8/136 | 20/619 | 1.202 | 0.813-1.776 | 55/89 | 217/422 | 1.068 | 0.61-1.855 | 20/124 | 84/556 |
| rs669607 | rs2744148 | no gene | SOX8 | 6 | CC | AG | 3.65*10^-04 E)^ | 3.187 | 1.658-6.099 | 19/125 | 29/608 | 1.602 | 1.068-2.402 | 50/94 | 159/479 | 1.544 | 1.034-2.302 | 52/92 | 171/467 |
| rs140522 | rs11154801 | SCO2 | MYB(AHI1) | 2 | AG | AA | 1.03*10^-01 E)^ | 1.999 | 0.865-4.526 | 10/134 | 23/616 | 1.439 | 0.985-2.101 | 75/69 | 275/364 | 1.068 | 0.61-1.855 | 20/124 | 84/556 |
| rs7200786 | rs9282641 | CLEC16A(CIITA) | CD86 | 4 | AA | AG | 1.67*10^-01 E)^ | 2.290 | 0.670-7.427 | 5/136 | 10/623 | 1.051 | 0.67-1.643 | 34/110 | 145/493 | 0.755 | 0.412-1.369 | 16/125 | 92/543 |
| rs10201872 | rs7200786 | SP140 | CLEC16A(CIITA) | 1 | *GG* | AA | 1.01*10^-01 E)^ | 1.522 | 0.902-2.557 | 25/119 | 77/558 | 1.082 | 0.728-1.609 | 94/50 | 405/233 | 1.051 | 0.67-1.643 | 34/110 | 145/493 |
| rs11154801 | rs2300603 | MYB(AHI1) | BATF | 3 | CC | GG | 1.00*10^0 E)^ | 0.000 | 0.00-76.792 | 0/144 | 1/634 | 0.726 | 0.487-1.08 | 48/96 | 261/379 | 0.708 | 0.284-1.684 | 7/137 | 43/596 |
| rs10466829 | rs7200786 | CLECL1 | CLEC16A(CIITA) | 9 | GG | GG | 1.70*10^-01 E)^ | 0.552 | 0.224-1.294 | 7/137 | 54/583 | 0.801 | 0.509-1.257 | 32/112 | 168/471 | 0.853 | 0.554-1.311 | 37/107 | 184/454 |
| rs180515 | rs228614 | RPS6KB1 | NFKB1(MANBA) | 9 | GG | GG | 2.49*10^-03 E)^ | 3.669 | 1.517-8.812 | 11/133 | 14/621 | 1.444 | 0.85-2.438 | 24/120 | 78/563 | 1.087 | 0.712-1.655 | 41/103 | 171/467 |
| rs7522462 | rs13192841 | C1orf106(KIF21B) | no gene | 3 | GG | AA | 3.43*10^-03 E)^ | 3.450 | 1.443-8.175 | 11/132 | 15/621 | 1.405 | 0.953-2.074 | 89/54 | 346/295 | 1.310 | 0.651-2.594 | 13/131 | 45/594 |
| rs11129295 | rs12368653 | EOMES | CYP27B1 | 7 | GG | GG | 1.00*10^0 E)^ | 0.988 | 0.550-1.756 | 18/126 | 80/553 | 1.022 | 0.692-1.508 | 55/89 | 240/397 | 1.027 | 0.676-1.558 | 42/102 | 182/454 |
| rs10466829 | rs10201872 | CLECL1 | SP140 | 6 | GG | *AG* | 8.77*10^-01 E)^ | 0.904 | 0.459-1.751 | 13/131 | 63/574 | 0.801 | 0.509-1.257 | 32/112 | 168/471 | 1.066 | 0.714-1.59 | 49/95 | 208/430 |

**Table B in S1. p-value and ORs for genotype/genotype combinations for model A).** p-value and ORs for genotype/genotype combinations, which passed the selection criteria, calculated using model A) in comparison to the underlying single genotypes ORs present in that given combination. column 1 = SNP1; column 2 = SNP2; column 3 and 4 = suggested genes by IMSGC and WTCCC2 for SNP1 and SNP2; column 5 = recoding risk factor according to figure 5; column 6 and 7 = genotypes included in the recoding stated in column 5 –risk alleles for the corresponding genes are underlined (italic = deviation of previously identified risk alleles); column 8 = p-value for the genotype/genotype combination; column 9= odds ratio (OR) for that given genotype/genotype combination; column 10 = 95% confidence interval (C.I.); column 11 = number of patients – left of the diagonal slash equals to the patients with the risk combination / right of the diagonal slash equals to the remaining eight combinations; column 12= analogous to column 11 only for controls; column 13 = OR for the underlying single genotype at SNP locus 1; 14 = 95% C.I.; columns 15 and 16 = analogues to columns 11 and 12; columns 17 to 20 = analogues to columns 13 to 16 only for SNP 2. ^A)^ = passing FDR < 5%. ^B)^ = passing FDR < 10%. ^C)^ = passing FDR < 15%. ^D)^ = passing FDR < 20%. ^E)^ = FDR > 20%.

| Model B) |  |  |  |  |  |  |  |  |  |  |  |  |  |  |  |  |  |  |  |
| --- | --- | --- | --- | --- | --- | --- | --- | --- | --- | --- | --- | --- | --- | --- | --- | --- | --- | --- | --- |
| all MS vs. controls | | |  |  |  |  | SNP1-SNP2 combination | | | | | SNP1 | | | | SNP2 | | | |
| SNP1 | SNP2 | Gene 1 | Gene 2 | Coding | SNP1 | SNP2 | p val | OR | 95% C.I. | Pat | Contr | OR | 95% C.I. | Pat | Contr | OR | 95% C.I. | Pat | Contr |
| rs630923 | rs2293370 | CXCR5 | TMEM39A(CD80) | 9 | AA+AC | AA+AG | 6.39*10^-05 A)^ | 0.483 | 0.334-0.699 | 61/962 | 74/564 | 0.773 | 0.618-0.967 | 268/757 | 201/439 | 0.816 | 0.657-1.014 | 305/722 | 218/421 |
| rs10201872 | rs1738074 | SP140 | TAGAP | 1 | *GA*+*AA* | AG+AA | 5.03*10^-04 A)^ | 0.656 | 0.515-0.835 | 197/823 | 169/463 | 0.882 | 0.713-1.09 | 346/682 | 233/405 | 0.937 | 0.761-1.154 | 608/413 | 388/247 |
| rs2425752 | rs2744148 | CD40 | SOX8 | 1 | AG+AA | AG+GG | 1.11*10^-04 E)^ | 1.755 | 1.302-2.368 | 192/828 | 74/560 | 1.137 | 0.928-1.394 | 506/516 | 294/341 | 1.303 | 1.045-1.625 | 351/675 | 182/456 |
|  |  |  |  |  |  |  |  |  |  |  |  |  |  |  |  |  |  |  |  |
| RR/SPMS+CIS vs. controls | | |  |  |  |  | SNP1-SNP2 combination | | | | | SNP1 | | | | SNP2 | | | |
| SNP1 | SNP2 | Gene 1 | Gene 2 | Coding | SNP1 | SNP2 | p val | OR | 95% C.I. | Pat | Contr | OR | 95% C.I. | Pat | Contr | OR | 95% C.I. | Pat | Contr |
| rs630923 | rs2293370 | CXCR5 | TMEM39A(CD80) | 9 | AA+AC | AA+AG | 4.94*10^-05 A)^ | 0.454 | 0.305-0.676 | 47/789 | 74/564 | 0.764 | 0.605-0.966 | 217/620 | 201/439 | 0.791 | 0.63-0.993 | 244/596 | 218/421 |
| rs10201872 | rs1738074 | SP140 | TAGAP | 1 | *GA*+*AA* | AG+AA | 1.30*10^-04 A)^ | 0.615 | 0.476-0.794 | 153/682 | 169/463 | 0.877 | 0.703-1.094 | 282/559 | 233/405 | 0.913 | 0.735-1.134 | 492/343 | 388/247 |
| rs2425752 | rs2744148 | CD40 | SOX8 | 1 | AG+AA | AG+GG | 1.26*10^-04 A)^ | 1.785 | 1.312-2.431 | 159/674 | 74/560 | 1.157 | 0.936-1.431 | 417/418 | 294/341 | 1.298 | 1.032-1.634 | 286/552 | 182/456 |
|  |  |  |  |  |  |  |  |  |  |  |  |  |  |  |  |  |  |  |  |
| PPMS vs. controls | | |  |  |  |  | SNP1-SNP2 combination | | | | | SNP1 | | | | SNP2 | | | |
| SNP1 | SNP2 | Gene 1 | Gene 2 | Coding | SNP1 | SNP2 | p val | OR | 95% C.I. | Pat | Contr | OR | 95% C.I. | Pat | Contr | OR | 95% C.I. | Pat | Contr |
| rs630923 | rs2293370 | CXCR5 | TMEM39A(CD80) | 9 | AA+AC | AA+AG | 1.35*10^-01 E)^ | 0.569 | 0.268-1.172 | 10/134 | 74/564 | 0.811 | 0.531-1.237 | 39/105 | 201/439 | 0.906 | 0.604-1.357 | 46/98 | 218/421 |
| rs10201872 | rs1738074 | SP140 | TAGAP | 1 | *GA*+*AA* | AG+AA | 4.00*10^-01 E)^ | 0.822 | 0.524-1.285 | 33/110 | 169/463 | 0.925 | 0.622-1.373 | 50/94 | 233/405 | 1.019 | 0.69-1.505 | 88/55 | 388/247 |
| rs2425752 | rs2744148 | CD40 | SOX8 | 1 | AG+AA | AG+GG | 1.00*10^0 E)^ | 0.973 | 0.542-1.728 | 18/125 | 82/554 | 1.160 | 0.795-1.693 | 72/72 | 294/341 | 1.503 | 1.011-2.233 | 54/90 | 182/456 |

**Table C in S1. p-value and ORs for genotype/genotype combinations for model B).** p-value and ORs for genotype/genotype combinations, which passed the selection criteria, calculated using model B) in comparison to the underlying single genotypes ORs present in that given combination. column 1 = SNP1; column 2 = SNP2; column 3 and 4 = suggested genes by IMSGC and WTCCC2 for SNP1 and SNP2; column 5 = recoding risk factor according to figure 5; column 6 and 7 = genotypes included in the recoding stated in column 5 –risk alleles for the corresponding genes are underlined (italic = deviation of previously identified risk alleles); column 8 = p-value for the genotype/genotype combination; column 9= odds ratio (OR) for that given genotype/genotype combination; column 10 = 95% confidence interval (C.I.); column 11 = number of patients – left of the diagonal slash equals to the patients with the risk combination / right of the diagonal slash equals to the remaining eight combinations; column 12= analogous to column 11 only for controls; column 13 = OR for the underlying single genotype at SNP locus 1; 14 = 95% C.I.; columns 15 and 16 = analogues to columns 11 and 12; columns 17 to 20 = analogues to columns 13 to 16 only for SNP 2. ^A)^ = passing FDR < 5%. ^B)^ = passing FDR < 10%. ^C)^ = passing FDR < 15%. ^D)^ = passing FDR < 20%. ^E)^ = FDR > 20%.

| Model C) |  |  |  |  |  |  |  |  |  |  |  |  |  |  |  |  |  |  |  |
| --- | --- | --- | --- | --- | --- | --- | --- | --- | --- | --- | --- | --- | --- | --- | --- | --- | --- | --- | --- |
| all MS vs. controls | | |  |  |  |  | SNP1-SNP2 combination | | | | | SNP1 | | | | SNP2 | | | |
| SNP1 | SNP2 | Gene 1 | Gene 2 | Coding | SNP1 | SNP2 | p val | OR | 95% C.I. | Pat | Contr | OR | 95% C.I. | Pat | Contr | OR | 95% C.I. | Pat | Contr |
| rs13192841 | rs1800693 | no gene | TNFRSF1A | 3 | AA+AG | GG | 1.92*10^-05 A)^ | 2.180 | 1.485-3.209 | 130/890 | 40/597 | 1.485 | 1.21-1.822 | 520/504 | 262/377 | 1.290 | 1.001-1.664 | 234/791 | 119/519 |
| rs630923 | rs140522 | CXCR5 | SCO2 | 9 | AA+AC | GG | 1.16*10^-04 B)^ | 0.551 | 0.404-0.753 | 95/927 | 100/538 | 0.773 | 0.618-0.967 | 268/757 | 201/439 | 0.759 | 0.619-0.931 | 417/609 | 303/336 |
| rs2744148 | rs2019960 | SOX8 | PVT1 | 1 | AG+GG | GG | 7.69*10^-04 B)^ | 7.624 | 1.744-46.823 | 24/1001 | 2/636 | 1.303 | 1.045-1.625 | 351/675 | 182/456 | 1.874 | 1.04-3.411 | 50/978 | 17/623 |
| rs2028597 | rs669607 | CBLB | no gene | 3 | AA+AG | AA | 1.08*10^-03 B)^ | 0.403 | 0.225-0.719 | 22/998 | 33/603 | 0.747 | 0.562-0.993 | 135/885 | 108/529 | 0.699 | 0.552-0.885 | 215/814 | 175/463 |
| rs2028597 | rs630923 | CBLB | CXCR5 | 6 | AA+AG | AC | 1.23*10^-03 B)^ | 0.457 | 0.276-0.755 | 31/985 | 41/595 | 0.747 | 0.562-0.993 | 135/885 | 108/529 | 0.699 | 0.552-0.885 | 215/814 | 175/463 |
| rs2119704 | rs12368653 | GALC(GPR65) | CYP27B1 | 9 | AA+AC | GG | 5.65*10^-03 D)^ | 0.507 | 0.305-0.841 | 32/992 | 38/597 | 0.837 | 0.629-1.114 | 141/885 | 102/536 | 0.837 | 0.557-1.259 | 64/963 | 47/592 |
| rs2019960 | rs4648356 | PVT1 | MMEL1 (TNFRSF14) | 3 | GG+GA | *AA* | 5.83*10^-03 D)^ | 2.673 | 1.233-5.978 | 38/984 | 9/623 | 1.229 | 0.995-1.519 | 401/627 | 219/421 | 0.837 | 0.666-1.052 | 258/769 | 182/454 |
| rs1077667 | rs2300603 | TNFSF14 | BATF | 1 | AG+AA | GG | 6.34*10^-03 D)^ | 0.398 | 0.196-0.801 | 15/1007 | 23/614 | 0.739 | 0.598-0.914 | 327/698 | 249/393 | 1.008 | 0.677-1.502 | 75/948 | 46/586 |
| rs12466022 | rs1250550 | no gene | ZMIZ1 | 3 | AA+AC | *AA* | 8.44*10^-03 D)^ | 0.539 | 0.334-0.871 | 37/981 | 41/586 | 0.841 | 0.686-1.031 | 443/582 | 304/336 | 0.959 | 0.698-1.318 | 119/903 | 76/553 |
| rs9282641 | rs1323292 | CD86 | RGS1 | 6 | AA+AG | AG | 9.29*10^-03 D)^ | 0.527 | 0.32-0.865 | 34/980 | 39/592 | 1.017 | 0.768-1.348 | 161/857 | 99/536 | 1.002 | 0.801-1.254 | 295/728 | 184/455 |
| rs2119704 | rs12212193 | GALC(GPR65) | BACH2 | 9 | AA+AC | *GG* | 9.63*10^-03 D)^ | 0.495 | 0.286-0.856 | 27/998 | 33/604 | 0.837 | 0.629-1.114 | 141/885 | 102/536 | 0.820 | 0.645-1.042 | 221/807 | 160/479 |
| rs17174870 | rs650258 | MERTK | CD6 | 7 | AG+AA | AA | 1.03*10^-02 D)^ | 0.531 | 0.325-0.866 | 35/985 | 40/598 | 0.921 | 0.748-1.134 | 387/637 | 254/385 | 0.938 | 0.688-1.277 | 124/900 | 82/558 |
| rs7238078 | rs2303759 | MALT1 | DKKL1(CD37) | 3 | *AC+CC* | CC | 1.19*10^-02 D)^ | 2.490 | 1.141-5.603 | 35/981 | 9/628 | 0.999 | 0.81-1.231 | 380/639 | 240/403 | 1.209 | 0.792-1.851 | 73/953 | 38/600 |
| rs2293370 | rs4613763 | TMEM39A(CD80) | PTGER4 | 3 | AA+AG | *GG* | 1.39*10^-02 D)^ | 0.205 | 0.044-0.826 | 3/1022 | 9/629 | 0.816 | 0.657-1.014 | 305/722 | 218/421 | 0.814 | 0.403-1.649 | 21/1005 | 16/623 |
| rs2019960 | rs669607 | PVT1 | no gene | 6 | AG+GG | AC | 6.41*10^-04 E)^ | 1.603 | 1.213-2.119 | 212/816 | 89/549 | 1.229 | 0.995-1.519 | 401/627 | 219/421 | 1.167 | 0.953-1.429 | 530/499 | 304/334 |
| rs630923 | rs2248359 | CXCR5 | CYP24A1^*^ | 9 | AA+AC | AA | 2.08*10^-02 E)^ | 0.532 | 0.307-0.918 | 28/994 | 32/604 | 0.773 | 0.618-0.967 | 268/757 | 201/439 | 0.837 | 0.64-1.095 | 164/862 | 118/519 |
| rs1335532 | rs1800693 | CD58 | TNFRSF1A | 7 | AG+GG | AA | 4.00*10^-03 E)^ | 0.557 | 0.37-0.838 | 52/969 | 56/581 | 0.808 | 0.632-1.032 | 205/820 | 152/491 | 0.847 | 0.683-1.05 | 319/706 | 222/416 |
|  |  |  |  |  |  |  |  |  |  |  |  |  |  |  |  |  |  |  |  |
| RR/SPMS+CIS vs. controls | | |  |  |  |  | SNP1-SNP2 combination | | | | | SNP1 | | | | SNP2 | | | |
| SNP1 | SNP2 | Gene 1 | Gene 2 | Coding | SNP1 | SNP2 | p val | OR | 95% C.I. | Pat | Contr | OR | 95% C.I. | Pat | Contr | OR | 95% C.I. | Pat | Contr |
| rs13192841 | rs1800693 | no gene | TNFRSF1A | 3 | AA+AG | GG | 1.66*10^-05 A)^ | 2.247 | 1.516-3.34 | 109/724 | 40/597 | 1.550 | 1.252-1.918 | 434/403 | 262/377 | 1.307 | 1.004-1.703 | 193/644 | 119/519 |
| rs630923 | rs140522 | CXCR5 | SCO2 | 9 | AA+AC | GG | 1.90*10^-04 B)^ | 0.547 | 0.393-0.759 | 77/758 | 100/538 | 0.764 | 0.605-0.966 | 217/620 | 201/439 | 0.794 | 0.642-0.982 | 350/489 | 303/336 |
| rs2744148 | rs2019960 | SOX8 | PVT1 | 1 | AG+GG | GG | 4.50*10^-04 B)^ | 8.184 | 1.851-50.679 | 21/816 | 2/636 | 1.298 | 1.032-1.634 | 286/552 | 182/456 | 1.977 | 1.083-3.646 | 43/797 | 17/623 |
| rs2028597 | rs669607 | CBLB | no gene | 3 | AA+AG | AA | 4.67*10^-03 E)^ | 0.447 | 0.244-0.812 | 20/818 | 33/603 | 0.803 | 0.598-1.077 | 118/720 | 108/529 | 0.720 | 0.563-0.922 | 180/661 | 175/463 |
| rs2028597 | rs630923 | CBLB | CXCR5 | 6 | AA+AG | AC | 6.12*10^-03 E)^ | 0.504 | 0.3-0.846 | 28/806 | 41/595 | 0.803 | 0.598-1.077 | 118/720 | 108/529 | 0.759 | 0.596-0.966 | 195/642 | 183/457 |
| rs2119704 | rs12368653 | GALC(GPR65) | CYP27B1 | 9 | AA+AC | GG | 9.37*10^-03 E)^ | 0.502 | 0.292-0.859 | 26/814 | 38/597 | 0.866 | 0.643-1.166 | 119/722 | 102/536 | 0.816 | 0.642-1.037 | 207/633 | 182/454 |
| rs2019960 | rs4648356 | PVT1 | MMEL1 (TNFRSF14) | 3 | GG+GA | *AA* | 4.06*10^-03 D)^ | 2.845 | 1.295-6.438 | 33/803 | 9/623 | 1.288 | 1.034-1.604 | 337/503 | 219/421 | 1.055 | 0.699-1.593 | 64/773 | 46/586 |
| rs1077667 | rs2300603 | TNFSF14 | BATF | 1 | AG+AA | GG | 3.20*10^-02 E)^ | 0.489 | 0.241-0.986 | 15/819 | 23/614 | 0.715 | 0.573-0.893 | 261/576 | 249/393 | 0.824 | 0.526-1.289 | 47/791 | 43/596 |
| rs12466022 | rs1250550 | no gene | ZMIZ1 | 3 | AA+AC | *AA* | 8.97*10^-03 E)^ | 0.516 | 0.308-0.862 | 29/803 | 41/586 | 0.857 | 0.693-1.06 | 366/472 | 304/336 | 0.923 | 0.661-1.29 | 94/741 | 76/553 |
| rs9282641 | rs1323292 | CD86 | RGS1 | 6 | AA+AG | AG | 2.49*10^-02 E)^ | 0.569 | 0.34-0.951 | 30/800 | 39/592 | 1.047 | 0.782-1.403 | 135/698 | 99/536 | 0.984 | 0.697-1.39 | 91/745 | 69/556 |
| rs2119704 | rs12212193 | GALC(GPR65) | BACH2 | 9 | AA+AC | *GG* | 7.61*10^-03 E)^ | 0.469 | 0.259-0.846 | 21/819 | 33/604 | 0.866 | 0.643-1.166 | 119/722 | 102/536 | 1.031 | 0.817-1.302 | 246/590 | 184/455 |
| rs17174870 | rs650258 | MERTK | CD6 | 7 | AG+AA | AA | 2.69*10^-02 E)^ | 0.577 | 0.347-0.957 | 31/803 | 40/598 | 0.904 | 0.727-1.123 | 313/525 | 254/385 | 0.765 | 0.594-0.985 | 171/669 | 160/479 |
| rs7238078 | rs2303759 | MALT1 | DKKL1(CD37) | 3 | *AC+CC* | CC | 8.82*10^-03 E)^ | 2.711 | 1.227-6.168 | 31/798 | 9/628 | 1.018 | 0.818-1.266 | 314/518 | 240/403 | 1.196 | 0.769-1.862 | 59/779 | 38/600 |
| rs2293370 | rs4613763 | TMEM39A(CD80) | PTGER4 | 3 | AA+AG | *GG* | 1.25*10^-02 E)^ | 0.167 | 0.025-0.828 | 2/836 | 9/629 | 0.791 | 0.63-0.993 | 244/596 | 218/421 | 0.952 | 0.468-1.945 | 20/818 | 16/623 |
| rs2019960 | rs669607 | PVT1 | no gene | 6 | AG+GG | AC | 8.46*10^-05 A)^ | 1.742 | 1.309-2.321 | 185/655 | 89/549 | 1.288 | 1.034-1.604 | 337/503 | 219/421 | 1.217 | 0.985-1.504 | 442/399 | 304/334 |
| rs630923 | rs2248359 | CXCR5 | CYP24A1^*^ | 9 | AA+AC | AA | 3.35*10^-03 D)^ | 0.416 | 0.222-0.774 | 18/817 | 32/604 | 0.764 | 0.605-0.966 | 217/620 | 201/439 | 0.806 | 0.608-1.07 | 130/709 | 118/519 |
| rs1335532 | rs1800693 | CD58 | TNFRSF1A | 7 | AG+GG | AA | 4.01*10^-03 D)^ | 0.537 | 0.347-0.831 | 41/792 | 56/581 | 0.817 | 0.633-1.056 | 169/668 | 152/491 | 0.844 | 0.674-1.058 | 260/577 | 222/416 |
|  |  |  |  |  |  |  |  |  |  |  |  |  |  |  |  |  |  |  |  |
| PPMS vs. controls | | |  |  |  |  | SNP1-SNP2 combination | | | | | SNP1 | | | | SNP2 | | | |
| SNP1 | SNP2 | Gene 1 | Gene 2 | Coding | SNP1 | SNP2 | p val | OR | 95% C.I. | Pat | Contr | OR | 95% C.I. | Pat | Contr | OR | 95% C.I. | Pat | Contr |
| rs13192841 | rs1800693 | no gene | TNFRSF1A | 3 | AA+AG | GG | 1.38*10^-02 E)^ | 2.132 | 1.134-3.978 | 18/126 | 40/597 | 1.287 | 0.881-1.881 | 68/76 | 262/377 | 1.297 | 0.818-2.049 | 33/111 | 119/519 |
| rs630923 | rs140522 | CXCR5 | SCO2 | 9 | AA+AC | GG | 1.17*10^-02 E)^ | 0.445 | 0.219-0.882 | 11/133 | 100/538 | 0.811 | 0.531-1.237 | 39/105 | 201/439 | 0.554 | 0.372-0.824 | 48/96 | 303/336 |
| rs2744148 | rs2019960 | SOX8 | PVT1 | 1 | AG+GG | GG | 1.57*10^-01 E)^ | 4.479 | 0.448-44.795 | 2/142 | 2/636 | 1.503 | 1.011-2.233 | 54/90 | 182/456 | 1.047 | 0.293-3.374 | 4/140 | 17/623 |
| rs2028597 | rs669607 | CBLB | no gene | 3 | AA+AG | AA | 1.96*10^-03 E)^ | 0.000 | 0-0.636 | 0/143 | 33/603 | 0.490 | 0.254-0.927 | 13/130 | 108/529 | 0.529 | 0.321-0.867 | 24/120 | 175/463 |
| rs2028597 | rs630923 | CBLB | CXCR5 | 6 | AA+AG | AC | 1.39*10^-02 E)^ | 0.206 | 0.034-0.883 | 2/141 | 41/595 | 0.490 | 0.254-0.927 | 13/130 | 108/529 | 0.895 | 0.583-1.372 | 38/106 | 183/457 |
| rs2119704 | rs12368653 | GALC(GPR65) | CYP27B1 | 9 | AA+AC | GG | 5.47*10^-01 E)^ | 0.693 | 0.258-1.756 | 6/136 | 38/597 | 0.715 | 0.397-1.273 | 17/125 | 102/536 | 1.027 | 0.676-1.558 | 42/102 | 182/454 |
| rs2019960 | rs4648356 | PVT1 | MMEL1 (TNFRSF14) | 3 | GG+GA | *AA* | 4.69*10^-01 E)^ | 1.494 | 0.317-6.104 | 3/139 | 9/623 | 0.902 | 0.602-1.351 | 46/98 | 219/421 | 0.862 | 0.383-1.882 | 9/133 | 46/586 |
| rs1077667 | rs2300603 | TNFSF14 | BATF | 1 | AG+AA | GG | 1.29*10^-02 E)^ | 0.000 | 0-0.935 | 0/144 | 23/614 | 0.947 | 0.641-1.398 | 54/90 | 249/393 | 0.708 | 0.284-1.684 | 7/137 | 43/596 |
| rs12466022 | rs1250550 | no gene | ZMIZ1 | 3 | AA+AC | *AA* | 5.68*10^-01 E)^ | 0.741 | 0.297-1.768 | 7/135 | 41/586 | 0.799 | 0.545-1.171 | 60/83 | 304/336 | 1.323 | 0.765-2.272 | 22/121 | 76/553 |
| rs9282641 | rs1323292 | CD86 | RGS1 | 6 | AA+AG | AG | 1.54*10^-01 E)^ | 0.446 | 0.133-1.334 | 4/136 | 39/592 | 0.693 | 0.379-1.253 | 16/125 | 99/536 | 0.771 | 0.495-1.199 | 34/109 | 184/455 |
| rs2119704 | rs12212193 | GALC(GPR65) | BACH2 | 9 | AA+AC | *GG* | 1.81*10^-01 E)^ | 0.395 | 0.095-1.37 | 3/139 | 33/604 | 0.715 | 0.397-1.273 | 17/125 | 102/536 | 1.035 | 0.669-1.597 | 37/107 | 160/479 |
| rs17174870 | rs650258 | MERTK | CD6 | 7 | AG+AA | AA | 1.12*10^-01 E)^ | 0.430 | 0.129-1.283 | 4/139 | 40/598 | 1.005 | 0.682-1.479 | 57/86 | 254/385 | 0.911 | 0.501-1.637 | 17/127 | 82/558 |
| rs7238078 | rs2303759 | MALT1 | DKKL1(CD37) | 3 | *AC+CC* | CC | 2.72*10^-01 E)^ | 2.008 | 0.513-7.252 | 4/139 | 9/628 | 0.903 | 0.607-1.341 | 50/93 | 240/403 | 1.435 | 0.689-2.939 | 12/132 | 38/600 |
| rs2293370 | rs4613763 | TMEM39A(CD80) | PTGER4 | 3 | AA+AG | *GG* | 6.98*10^-01 E)^ | 0.489 | 0.023-3.793 | 1/143 | 9/629 | 0.906 | 0.604-1.357 | 46/98 | 218/421 | 0.272 | 0.013-1.967 | 1/143 | 16/623 |
| rs2019960 | rs669607 | PVT1 | no gene | 6 | AG+GG | AC | 6.92*10^-01 E)^ | 1.112 | 0.649-1.894 | 22/122 | 89/549 | 0.902 | 0.602-1.351 | 46/98 | 219/421 | 1.039 | 0.712-1.516 | 70/74 | 304/334 |
| rs630923 | rs2248359 | CXCR5 | CYP24A1^*^ | 9 | AA+AC | AA | 8.34*10^-01 E)^ | 1.110 | 0.461-2.585 | 8/136 | 32/604 | 0.811 | 0.531-1.237 | 39/105 | 201/439 | 1.015 | 0.621-1.651 | 27/117 | 118/519 |
| rs1335532 | rs1800693 | CD58 | TNFRSF1A | 7 | AG+GG | AA | 8.58*10^-02 E)^ | 0.451 | 0.171-1.117 | 6/138 | 56/581 | 0.679 | 0.413-1.108 | 25/119 | 152/491 | 0.825 | 0.547-1.239 | 44/100 | 222/416 |

**Table D in S1. p-value and ORs for genotype/genotype combinations for model C).** p-value and ORs for genotype/genotype combinations, which passed the selection criteria, calculated using model C) in comparison to the underlying single genotypes ORs present in that given combination. column 1 = SNP1; column 2 = SNP2; column 3 and 4 = suggested genes by IMSGC and WTCCC2 for SNP1 and SNP2; column 5 = recoding risk factor according to figure 5; column 6 and 7 = genotypes included in the recoding stated in column 5 –risk alleles for the corresponding genes are underlined (italic = deviation of previously identified risk alleles); column 8 = p-value for the genotype/genotype combination; column 9= odds ratio (OR) for that given genotype/genotype combination; column 10 = 95% confidence interval (C.I.); column 11 = number of patients – left of the diagonal slash equals to the patients with the risk combination / right of the diagonal slash equals to the remaining eight combinations; column 12= analogous to column 11 only for controls; column 13 = OR for the underlying single genotype at SNP locus 1; 14 = 95% C.I.; columns 15 and 16 = analogues to columns 11 and 12; columns 17 to 20 = analogues to columns 13 to 16 only for SNP 2. ^A)^ = passing FDR < 5%. ^B)^ = passing FDR < 10%. ^C)^ = passing FDR < 15%. ^D)^ = passing FDR < 20%. ^E)^ = FDR > 20%. ^*^ = HWE deviation of the controls.

|  |  |  | Ataxia in Pat (n_max_=545) | | | | | | | | | | | | | | | | | |  | Fam. MS in Pat (n_max_=372) | | | | | | | | | | | | | | |
| --- | --- | --- | --- | --- | --- | --- | --- | --- | --- | --- | --- | --- | --- | --- | --- | --- | --- | --- | --- | --- | --- | --- | --- | --- | --- | --- | --- | --- | --- | --- | --- | --- | --- | --- | --- | --- |
|  |  |  | genotypes | | | | | | | | | | | |  |  | |  | |  |  | genotypes | | | | | | | | | | |  |  |  |  |
|  |  |  | present | | | | | | absent | | | | | |  |  | |  | |  |  | present | | | | | absent | | | | | |  |  |  |  |
| SNP | Gene | allele-coding 1/2 | 11 | | 12 | | 22 | | 11 | | 12 | | 22 | | risk allel | p val | | OR | | 95% C.I. |  | 11 | 12 | | 22 | | 11 | | 12 | | 22 | | risk allel | p val | OR | 95% C.I. |
| rs949143 | ARL6IP4 | A/G | 195 | | 173 | | 37 | | 59 | | 68 | | 12 | | 1 | 0.41936 | | 1.127 | | 0.843-1.508 |  | 20 | 22 | | 6 | | 154 | | 142 | | 27 | | 2 | 0.31565 | 1.259 | 0.802-1.976 |
| rs12212193 | BACH2 | A/G | 130 | | 198 | | 78 | | 41 | | 67 | | 31 | | 1 | 0.41614 | | 1.12 | | 0.852-1.472 |  | 10 | 29 | | 9 | | 106 | | 152 | | 66 | | 2 | 0.34510 | 1.229 | 0.800-1.888 |
| rs2300603 | BATF | G/A | 23 | | 146 | | 236 | | 8 | | 54 | | 76 | | 2 | 0.57806 | | 1.094 | | 0.798-1.500 |  | 4 | 13 | | 31 | | 20 | | 120 | | 182 | | 2 | 0.52772 | 1.181 | 0.705-1.978 |
| rs7522462 | C1orf106(KIF21B) | A/G | 12 | | 133 | | 49 | | 22 | | 242 | | 82 | | 2 | 0.78142^e)^ | | 1.036 | | 0.805-1.335 |  | 3 | 14 | | 31 | | 23 | | 113 | | 187 | | 2 | 0.41929 | 1.241 | 0.735-2.095 |
| rs2028597 | CBLB | A/G | 0 | | 63 | | 343 | | 1 | | 21 | | 116 | | 2 | 0.75982 | | 1.081 | | 0.657-1.779 |  | 0 | 7 | | 41 | | 1 | | 50 | | 273 | | 2 | 0.80409 | 1.109 | 0.489-2.518 |
| rs650258 | CD6 | G/A | 181 | | 173 | | 49 | | 49 | | 71 | | 19 | | 1 | 0.09235 | | 1.273 | | 0.961-1.688 |  | 24 | 19 | | 5 | | 130 | | 151 | | 42 | | 1 | 0.23876 | 1.321 | 0.830-2.101 |
| rs2425752 | CD40 | G/A | 198 | | 170 | | 36 | | 73 | | 48 | | 17 | | 2 | 0.93997 | | 1.012 | | 0.750-1.364 |  | 20 | 24 | | 4 | | 157 | | 133 | | 31 | | 2 | 0.55785 | 1.146 | 0.726-1.809 |
| rs1335532 | CD58 | A/G | 317 | | 85 | | 1 | | 115 | | 23 | | 1 | | 2 | 0.39487 | | 1.225 | | 0.767-1.954 |  | 36 | 11 | | 1 | | 260 | | 62 | | 1 | | 2 | 0.27591 | 1.424 | 0.752-2.699 |
| rs9282641 | CD86 | A/G | 1 | | 69 | | 336 | | 0 | | 23 | | 116 | | 1 | 0.80941 | | 1.062 | | 0.650-1.736 |  | 0 | 11 | | 37 | | 1 | | 46 | | 277 | | 1 | 0.17042 | 1.618 | 0.809-3.236 |
| rs7200786 | **CLEC16A(CIITA)^a)^** | A/G | 121 | | 191 | | 94 | | 31 | | 66 | | 42 | | 1 | **0.03601^a)^** | | 1.339 | | 1.019-1.759 |  | 14 | 21 | | 13 | | 95 | | 146 | | 83 | | 2 | 0.88214 | 1.033 | 0.673-1.586 |
| rs10466829 | CLECL1 | A/G | 110 | | 205 | | 90 | | 37 | | 75 | | 27 | | 2 | 0.74515 | | 1.046 | | 0.796-1.375 |  | 13 | 23 | | 12 | | 85 | | 171 | | 68 | | 2 | 0.77211 | 1.065 | 0.694-1.636 |
| rs630923 | CXCR5 | A/C | 9 | | 96 | | 298 | | 6 | | 29 | | 104 | | 2 | 0.80399 | | 1.05 | | 0.714-1.545 |  | 0 | 11 | | 37 | | 11 | | 71 | | 240 | | 2 | 0.43279 | 1.304 | 0.670-2.537 |
| rs2248359 | CYP24A1 | G/A | 157 | | 180 | | 69 | | 58 | | 57 | | 23 | | 2 | 0.58692 | | 1.081 | | 0.816-1.433 |  | 15 | 22 | | 11 | | 124 | | 142 | | 57 | | 2 | 0.24773 | 1.289 | 0.837-1.984 |
| rs12368653 | CYP27B1 | A/G | 103 | | 210 | | 92 | | 36 | | 61 | | 42 | | 1 | 0.31165 | | 1.151 | | 0.876-1.512 |  | 15 | 24 | | 9 | | 80 | | 166 | | 77 | | 1 | 0.28998 | 1.262 | 0.820-1.943 |
| rs2303759 | DKKL1(CD37) | C/A | 30 | | 159 | | 215 | | 6 | | 57 | | 76 | | 1 | 0.45684 | | 1.126 | | 0.823-1.541 |  | 2 | 20 | | 26 | | 23 | | 136 | | 163 | | 2 | 0.50605 | 1.182 | 0.722-1.934 |
| rs11129295 | EOMES | G/A | 139 | | 195 | | 67 | | 52 | | 72 | | 14 | | 2 | 0.16078 | | 1.224 | | 0.923-1.624 |  | 17 | 26 | | 5 | | 105 | | 160 | | 56 | | 1 | 0.36702 | 1.225 | 0.788-1.906 |
| rs11810217 | EVI5 | G/A | 226 | | 144 | | 33 | | 74 | | 56 | | 8 | | 1 | 0.99156 | | 1.002 | | 0.734-1.368 |  | 26 | 20 | | 2 | | 183 | | 113 | | 27 | | 1 | 0.8587 | 1.046 | 0.638-1.715 |
| rs2119704 | GALC(GPR65) | A/C | 3 | | 43 | | 359 | | 2 | | 15 | | 122 | | 2 | 0.64076 | | 1.139 | | 0.659-1.971 |  | 0 | 4 | | 44 | | 1 | | 38 | | 284 | | 2 | 0.43307 | 1.518 | 0.531-4.342 |
| rs7923837 | HHEX | A/G | 43 | | 171 | | 190 | | 16 | | 64 | | 59 | | 2 | 0.40268 | | 1.131 | | 0.848-1.509 |  | 5 | 22 | | 21 | | 42 | | 136 | | 145 | | 2 | 0.88909 | 1.033 | 0.656-1.627 |
| rs3135388 | HLA | T/C | 25 | | 176 | | 205 | | 11 | | 71 | | 57 | | 2 | 0.07544 | | 1.303 | | 0.973-1.747 |  | 7 | 21 | | 20 | | 18 | | 139 | | 167 | | 1 | 0.05482 | 1.551 | 0.989-2.433 |
| rs3118470 | IL2RA | G/A | 44 | | 183 | | 176 | | 13 | | 56 | | 70 | | 1 | 0.20551 | | 1.211 | | 0.900-1.628 |  | 6 | 18 | | 24 | | 38 | | 146 | | 139 | | 2 | 0.54758 | 1.152 | 0.726-1.827 |
| rs1520333 | IL7 | A/G | 214 | | 161 | | 29 | | 72 | | 55 | | 12 | | 1 | 0.67208 | | 1.068 | | 0.788-1.446 |  | 21 | 24 | | 3 | | 176 | | 121 | | 27 | | 2 | 0.38509 | 1.229 | 0.772-1.956 |
| rs6897932 | **IL7R^a)^** | G/A | 221 | | 159 | | 20 | | 71 | | 58 | | 10 | | 1 | 0.29549 | | 1.178 | | 0.867-1.601 |  | 32 | 16 | | 0 | | 169 | | 133 | | 20 | | 1 | **0.03258^a)^** | 1.837 | 1.045-3.229 |
| rs2243123 | IL12A | G/A | 41 | | 164 | | 194 | | 7 | | 70 | | 62 | | 1 | 0.84905 | | 1.029 | | 0.765-1.385 |  | 5 | 20 | | 22 | | 29 | | 147 | | 145 | | 2 | 0.99743 | 1.001 | 0.629-1.592 |
| rs2546890 | IL12B | A/G | 133 | | 187 | | 85 | | 45 | | 68 | | 26 | | 2 | 0.79221 | | 1.038 | | 0.788-1.366 |  | 21 | 21 | | 6 | | 105 | | 150 | | 68 | | 1 | 0.06759 | 1.517 | 0.968-2.376 |
| rs17066096 | IL22RA2 | A/G | 201 | | 167 | | 37 | | 74 | | 51 | | 14 | | 2 | 0.67320 | | 1.067 | | 0.790-1.442 |  | 25 | 22 | | 1 | | 159 | | 130 | | 35 | | 1 | 0.24243 | 1.339 | 0.820-2.189 |
| rs13333054 | **IRF8^b)^** | G/A | 227 | | 161 | | 17 | | 77 | | 51 | | 11 | | 1 | 0.46568 | | 1.123 | | 0.822-1.534 |  | 35 | 11 | | 1 | | 173 | | 138 | | 13 | | 1 | **0.01468^b)^** | 2.111 | 1.145-3.893 |
| rs7238078 | MALT1 | C/A | 24 | | 136 | | 242 | | 6 | | 48 | | 84 | | 1 | 0.69435 | | 1.068 | | 0.768-1.486 |  | 1 | 23 | | 24 | | 19 | | 100 | | 202 | | 1 | 0.31657 | 1.286 | 0.785-2.106 |
| rs2283792 | MAPK1 | C/A | 124 | | 200 | | 82 | | 43 | | 66 | | 30 | | 1 | 0.88587 | | 1.02 | | 0.776-1.341 |  | 12 | 23 | | 13 | | 101 | | 160 | | 63 | | 2 | 0.20436 | 1.32 | 0.859-2.027 |
| rs17174870 | MERTK | G/A | 258 | | 122 | | 24 | | 91 | | 44 | | 4 | | 2 | 0.40509 | | 1.158 | | 0.820-1.636 |  | 32 | 14 | | 2 | | 205 | | 100 | | 18 | | 1 | 0.60371 | 1.156 | 0.669-1.995 |
| rs4648356 | MMEL1 (TNFRSF14) | A/C | 29 | | 177 | | 198 | | 13 | | 61 | | 63 | | 2 | 0.40393 | | 1.134 | | 0.844-1.526 |  | 1 | 21 | | 26 | | 25 | | 139 | | 157 | | 2 | 0.26832 | 1.324 | 0.804-2.180 |
| rs874628 | MPV17L2(IL12RB1) | A/G | 228 | | 156 | | 21 | | 75 | | 56 | | 8 | | 1 | 0.62800 | | 1.08 | | 0.790-1.477 |  | 30 | 18 | | 0 | | 174 | | 128 | | 21 | | 1 | 0.11177 | 1.548 | 0.900-2.660 |
| rs11154801 | MYB(AHII) | A/C | 69 | | 194 | | 143 | | 14 | | 79 | | 46 | | 1 | 0.48176 | | 1.105 | | 0.836-1.461 |  | 9 | 25 | | 14 | | 52 | | 149 | | 123 | | 1 | 0.28285 | 1.267 | 0.822-1.951 |
| rs4410871 | MYC | G/A | 229 | | 156 | | 21 | | 72 | | 55 | | 12 | | 1 | 0.1825 | | 1.231 | | 0.907-1.672 |  | 29 | 15 | | 4 | | 172 | | 129 | | 23 | | 1 | 0.52828 | 1.174 | 0.712-1.936 |
| rs228614 | NFKB1(MANBA) | A/G | 79 | | 212 | | 115 | | 36 | | 73 | | 30 | | 2 | 0.05742 | | 1.302 | | 0.991-1.711 |  | 8 | 29 | | 11 | | 79 | | 157 | | 88 | | 2 | 0.75074 | 1.072 | 0.698-1.647 |
| rs669607 | **no gene^a)^** | A/C | 98 | | 217 | | 91 | | 18 | | 79 | | 42 | | 2 | **0.00625^a)^** | | 1.467 | | 1.114-1.932 |  | 8 | 26 | | 14 | | 64 | | 184 | | 76 | | 2 | 0.42066 | 1.194 | 0.775-1.838 |
| rs12466022 | no gene | A/C | 33 | | 142 | | 230 | | 10 | | 53 | | 76 | | 2 | 0.84881 | | 1.031 | | 0.756-1.405 |  | 2 | 16 | | 30 | | 25 | | 125 | | 174 | | 2 | 0.19932 | 1.406 | 0.834-2.370 |
| rs13192841 | no gene | A/G | 38 | | 178 | | 189 | | 15 | | 62 | | 62 | | 2 | 0.59187 | | 1.083 | | 0.810-1.448 |  | 6 | 25 | | 17 | | 36 | | 133 | | 155 | | 1 | 0.17768 | 1.355 | 0.870-2.111 |
| rs7595037 | PLEK | G/A | 79 | | 200 | | 126 | | 23 | | 67 | | 48 | | 1 | 0.34594 | | 1.142 | | 0.866-1.507 |  | 10 | 26 | | 12 | | 53 | | 160 | | 110 | | 1 | 0.21193 | 1.314 | 0.855-2.020 |
| rs4613763 | PTGER4 | G/A | 7 | | 92 | | 307 | | 3 | | 29 | | 107 | | 1 | 0.8422 | | 1.042 | | 0.693-1.569 |  | 0 | 10 | | 38 | | 5 | | 67 | | 252 | | 2 | 0.67655 | 1.16 | 0.578-2.328 |
| rs2019960 | **PVT1^a)^** | G/A | 23 | | 149 | | 233 | | 5 | | 37 | | 97 | | 1 | **0.01316^a)^** | | 1.558 | | 1.095-2.217 |  | 1 | 16 | | 31 | | 20 | | 110 | | 193 | | 2 | 0.32886 | 1.31 | 0.761-2.258 |
| rs1323292 | RGS1 | A/G | 271 | | 120 | | 12 | | 94 | | 42 | | 3 | | 2 | 0.82129 | | 1.042 | | 0.728-1.493 |  | 32 | 16 | | 0 | | 213 | | 96 | | 14 | | 1 | 0.55466 | 1.188 | 0.671-2.103 |
| rs180515 | RPS6KB1 | A/G | 159 | | 182 | | 63 | | 48 | | 70 | | 21 | | 1 | 0.52183 | | 1.095 | | 0.829-1.447 |  | 13 | 25 | | 10 | | 116 | | 156 | | 52 | | 2 | 0.20939 | 1.317 | 0.856-2.025 |
| rs140522 | SCO2 | A/G | 55 | | 187 | | 164 | | 16 | | 58 | | 64 | | 1 | 0.23424 | | 1.192 | | 0.892-1.592 |  | 6 | 17 | | 25 | | 42 | | 148 | | 134 | | 2 | 0.28377 | 1.288 | 0.810-2.050 |
| rs2744148 | SOX8 | A/G | 270 | | 120 | | 15 | | 90 | | 45 | | 4 | | 1 | 0.84014 | | 1.036 | | 0.732-1.468 |  | 37 | 9 | | 2 | | 218 | | 95 | | 10 | | 1 | 0.30261 | 1.383 | 0.745-2.566 |
| rs10201872 | SP140 | G/A | 268 | | 123 | | 15 | | 92 | | 43 | | 4 | | 2 | 0.85449 | | 1.033 | | 0.728-1.468 |  | 32 | 16 | | 0 | | 220 | | 90 | | 14 | | 1 | 0.71346 | 1.113 | 0.628-1.974 |
| rs9891119 | STAT3 | A/C | 148 | | 199 | | 55 | | 51 | | 73 | | 13 | | 2 | 0.49756 | | 1.103 | | 0.830-1.467 |  | 15 | 25 | | 8 | | 116 | | 166 | | 38 | | 2 | 0.35784 | 1.226 | 0.794-1.894 |
| rs1738074 | TAGAP | G/A | 177 | | 165 | | 63 | | 60 | | 61 | | 16 | | 2 | 0.55275 | | 1.091 | | 0.818-1.456 |  | 22 | 18 | | 7 | | 146 | | 126 | | 52 | | 1 | 0.78322 | 1.066 | 0.676-1.682 |
| rs802734 | THEMIS | A/G | 214 | | 162 | | 27 | | 69 | | 52 | | 17 | | 1 | 0.16337 | | 1.236 | | 0.917-1.667 |  | 29 | 16 | | 3 | | 167 | | 129 | | 26 | | 1 | 0.28778 | 1.315 | 0.793-2.181 |
| rs2293370 | TMEM39A(CD80) | A/G | 13 | | 92 | | 301 | | 5 | | 33 | | 100 | | 2 | 0.67195 | | 1.085 | | 0.743-1.586 |  | 1 | 9 | | 38 | | 9 | | 78 | | 237 | | 2 | 0.38177 | 1.344 | 0.692-2.612 |
| rs1800693 | TNFRSF1A | G/A | 99 | | 178 | | 125 | | 29 | | 63 | | 47 | | 1 | 0.34993 | | 1.14 | | 0.866-1.500 |  | 9 | 19 | | 19 | | 78 | | 145 | | 100 | | 2 | 0.18841 | 1.344 | 0.864-2.091 |
| rs6062314 | TNFRSF6B | G/A | 3 | | 59 | | 344 | | 0 | | 23 | | 116 | | 2 | 0.88723 | | 1.037 | | 0.631-1.703 |  | 0 | 8 | | 40 | | 3 | | 45 | | 276 | | 1 | 0.87551 | 1.064 | 0.489-2.317 |
| rs1077667 | **TNFSF14^b)^** | G/A | 270 | | 113 | | 20 | | 94 | | 40 | | 4 | | 2 | 0.55741 | | 1.113 | | 0.778-1.591 |  | 27 | 15 | | 5 | | 228 | | 82 | | 13 | | 2 | **0.01979^b)^** | 1.805 | 1.092-2.982 |
| rs8112449 | TYK2(ICAM3) | A/G | 40 | | 181 | | 184 | | 14 | | 54 | | 70 | | 1 | 0.43813 | | 1.125 | | 0.836-1.514 |  | 6 | 21 | | 21 | | 34 | | 140 | | 149 | | 1 | 0.67086 | 1.103 | 0.702-1.734 |
| rs11581062 | VCAM1 | A/G | 209 | | 162 | | 35 | | 65 | | 65 | | 9 | | 1 | 0.68338 | | 1.064 | | 0.789-1.434 |  | 25 | 17 | | 6 | | 159 | | 137 | | 28 | | 2 | 0.93241 | 1.02 | 0.640-1.628 |
| rs4902647 | ZFP36L1 | G/A | 132 | | 184 | | 90 | | 42 | | 67 | | 30 | | 1 | 0.80448 | | 1.035 | | 0.787-1.361 |  | 20 | 19 | | 9 | | 98 | | 148 | | 78 | | 1 | 0.12444 | 1.409 | 0.908-2.186 |
| rs1250550 | ZMIZ1 | A/C | 42 | | 191 | | 169 | | 11 | | 60 | | 67 | | 1 | 0.17090 | | 1.23 | | 0.914-1.654 |  | 4 | 20 | | 23 | | 34 | | 144 | | 143 | | 2 | 0.53210 | 1.162 | 0.725-1.862 |
| rs354033 | ZNF746 | G/A | 218 | | 159 | | 28 | | 80 | | 52 | | 7 | | 2 | 0.35702 | | 1.161 | | 0.845-1.594 |  | 26 | 20 | | 2 | | 170 | | 130 | | 24 | | 1 | 0.61169 | 1.136 | 0.694-1.860 |
|  |  |  |  | |  | |  | |  | |  | |  | |  |  | |  | |  |  |  |  | |  | |  | |  | |  | |  |  |  |  |
|  |  |  | Paresis in Pat (n_max_=545) | | | | | | | | | | | | | | | | | |  | Impaired vision in Pat (n_max_=545) | | | | | | | | | | | | | | |
|  |  |  | genotypes | | | | | | | | | | |  | |  |  | |  | |  | genotypes | | | | | | | | | | |  |  |  |  |
|  |  |  | present | | | | | absent | | | | | |  | |  |  | |  | |  | present | | | | | | absent | | | | |  |  |  |  |
| SNP | Gene | allele-coding 1/2 | 11 | 12 | | 22 | | 11 | | 12 | | 22 | | risk allel | | p val | OR | | 95% C.I. | |  | 11 | | 12 | | 22 | | 11 | | 12 | | 22 | risk allel | p val | OR | 95% C.I. |
| rs949143 | ARL6IP4 | A/G | 186 | 179 | | 39 | | 68 | | 62 | | 10 | | 2 | | 0.43245 | 1.126 | | 0.837-1.516 | |  | 179 | | 165 | | 35 | | 75 | | 76 | | 14 | 1 | 0.86675 | 1.024 | 0.775-1.353 |
| rs12212193 | BACH2 | A/G | 122 | 207 | | 76 | | 49 | | 58 | | 33 | | 2 | | 0.99183 | 1.001 | | 0.762-1.316 | |  | 111 | | 191 | | 77 | | 60 | | 74 | | 32 | 2 | 0.22717 | 1.174 | 0.905-1.524 |
| rs2300603 | BATF | G/A | 25 | 152 | | 227 | | 6 | | 48 | | 85 | | 1 | | 0.25068 | 1.211 | | 0.873-1.680 | |  | 22 | | 139 | | 217 | | 9 | | 61 | | 95 | 1 | 0.92466 | 1.015 | 0.750-1.373 |
| rs7522462 | **C1orf106(KIF21B)^a)^** | A/G | 23 | 134 | | 245 | | 12 | | 48 | | 79 | | 2 | | 0.23250 | 1.212 | | 0.884-1.661 | |  | 22 | | 118 | | 237 | | 13 | | 64 | | 87 | 2 | **0.03321^a)^** | 1.382 | 1.025-1.862 |
| rs2028597 | **CBLB^b)^** | A/G | 1 | 62 | | 342 | | 0 | | 22 | | 117 | | 2 | | 0.99471 | 1.002 | | 0.605-1.659 | |  | 0 | | 68 | | 311 | | 1 | | 16 | | 148 | 1 | **0.04814^b)^** | 1.708 | 0.999-2.921 |
| rs650258 | CD6 | G/A | 181 | 173 | | 49 | | 49 | | 71 | | 19 | | 1 | | 0.09235 | 1.273 | | 0.961-1.688 | |  | 167 | | 166 | | 45 | | 63 | | 78 | | 23 | 1 | 0.21142 | 1.187 | 0.907-1.554 |
| rs2425752 | **CD40^a)^** | G/A | 195 | 162 | | 46 | | 76 | | 56 | | 7 | | 2 | | **0.04667^a)^** | 1.367 | | 1.004-1.862 | |  | 181 | | 156 | | 39 | | 90 | | 62 | | 14 | 2 | 0.18387 | 1.215 | 0.912-1.619 |
| rs1335532 | **CD58^a)^** | A/G | 324 | 78 | | 1 | | 108 | | 30 | | 1 | | 1 | | 0.45399 | 1.18 | | 0.764-1.823 | |  | 312 | | 65 | | 1 | | 120 | | 43 | | 1 | 1 | **0.01580^a)^** | 1.635 | 1.094-2.445 |
| rs9282641 | CD86 | A/G | 1 | 69 | | 335 | | 0 | | 23 | | 117 | | 1 | | 0.77702 | 1.074 | | 0.657-1.754 | |  | 0 | | 63 | | 316 | | 1 | | 29 | | 136 | 2 | 0.57865 | 1.136 | 0.724-1.783 |
| rs7200786 | **CLEC16A(CIITA)^a)^** | A/G | 118 | 191 | | 96 | | 34 | | 66 | | 40 | | 1 | | 0.16080 | 1.215 | | 0.925-1.594 | |  | 113 | | 186 | | 80 | | 39 | | 71 | | 56 | 1 | **0.00397^a)^** | 1.462 | 1.129-1.895 |
| rs10466829 | CLECL1 | A/G | 109 | 208 | | 87 | | 38 | | 72 | | 30 | | 2 | | 0.96904 | 1.005 | | 0.766-1.320 | |  | 95 | | 202 | | 82 | | 52 | | 78 | | 35 | 2 | 0.29662 | 1.148 | 0.886-1.488 |
| rs630923 | CXCR5 | A/C | 11 | 97 | | 296 | | 4 | | 28 | | 106 | | 1 | | 0.49014 | 1.151 | | 0.771-1.719 | |  | 10 | | 92 | | 277 | | 5 | | 33 | | 125 | 1 | 0.49407 | 1.141 | 0.782-1.666 |
| rs2248359 | CYP24A1 | G/A | 151 | 180 | | 73 | | 64 | | 57 | | 19 | | 2 | | 0.05741 | 1.317 | | 0.991-1.751 | |  | 144 | | 170 | | 64 | | 71 | | 67 | | 28 | 2 | 0.45990 | 1.106 | 0.847-1.443 |
| rs12368653 | CYP27B1 | A/G | 103 | 208 | | 93 | | 36 | | 63 | | 41 | | 1 | | 0.38322 | 1.129 | | 0.860-1.481 | |  | 100 | | 192 | | 86 | | 39 | | 79 | | 48 | 1 | 0.16573 | 1.2 | 0.927-1.554 |
| rs2303759 | DKKL1(CD37) | C/A | 24 | 160 | | 219 | | 12 | | 56 | | 72 | | 2 | | 0.36656 | 1.15 | | 0.849-1.558 | |  | 27 | | 149 | | 202 | | 9 | | 67 | | 89 | 1 | 0.70713 | 1.058 | 0.788-1.420 |
| rs11129295 | EOMES | G/A | 138 | 204 | | 59 | | 53 | | 63 | | 22 | | 2 | | 0.68589 | 1.06 | | 0.801-1.402 | |  | 132 | | 183 | | 59 | | 59 | | 84 | | 22 | 2 | 0.65335 | 1.063 | 0.815-1.386 |
| rs11810217 | EVI5 | G/A | 227 | 146 | | 31 | | 73 | | 54 | | 10 | | 1 | | 0.68025 | 1.067 | | 0.783-1.455 | |  | 206 | | 146 | | 25 | | 94 | | 54 | | 16 | 1 | 0.93828 | 1.012 | 0.753-1.359 |
| rs2119704 | GALC(GPR65) | A/C | 3 | 45 | | 356 | | 2 | | 13 | | 125 | | 1 | | 0.88610 | 1.042 | | 0.591-1.837 | |  | 1 | | 42 | | 335 | | 4 | | 16 | | 146 | 2 | 0.37671 | 1.261 | 0.753-2.110 |
| rs7923837 | HHEX | A/G | 49 | 169 | | 185 | | 10 | | 66 | | 64 | | 1 | | 0.45784 | 1.117 | | 0.833-1.498 | |  | 48 | | 158 | | 172 | | 11 | | 77 | | 77 | 1 | 0.24433 | 1.181 | 0.893-1.562 |
| rs3135388 | HLA | T/C | 31 | 165 | | 209 | | 5 | | 82 | | 53 | | 2 | | 0.12550^e)^ | 1.257 | | 0.938-1.684 | |  | 22 | | 166 | | 191 | | 14 | | 81 | | 71 | 2 | 0.08687 | 1.276 | 0.965-1.686 |
| rs3118470 | IL2RA | G/A | 41 | 179 | | 183 | | 16 | | 60 | | 63 | | 2 | | 0.82723 | 1.033 | | 0.773-1.380 | |  | 43 | | 167 | | 167 | | 14 | | 72 | | 79 | 1 | 0.29318 | 1.161 | 0.879-1.536 |
| rs1520333 | IL7 | A/G | 216 | 157 | | 31 | | 70 | | 59 | | 10 | | 1 | | 0.67208 | 1.068 | | 0.788-1.446 | |  | 193 | | 156 | | 29 | | 93 | | 60 | | 12 | 2 | 0.33261 | 1.156 | 0.862-1.551 |
| rs6897932 | IL7R | G/A | 220 | 162 | | 22 | | 72 | | 55 | | 8 | | 1 | | 0.79420 | 1.043 | | 0.762-1.427 | |  | 205 | | 148 | | 23 | | 87 | | 69 | | 7 | 2 | 0.90721 | 1.018 | 0.756-1.371 |
| rs2243123 | IL12A | G/A | 37 | 175 | | 187 | | 11 | | 59 | | 69 | | 1 | | 0.51995 | 1.103 | | 0.818-1.487 | |  | 33 | | 163 | | 177 | | 15 | | 71 | | 79 | 1 | 0.97619 | 1.004 | 0.758-1.330 |
| rs2546890 | IL12B | A/G | 132 | 184 | | 88 | | 46 | | 71 | | 23 | | 2 | | 0.42103 | 1.12 | | 0.850-1.474 | |  | 122 | | 180 | | 76 | | 56 | | 75 | | 35 | 2 | 0.94128 | 1.01 | 0.779-1.310 |
| rs17066096 | IL22RA2 | A/G | 201 | 165 | | 38 | | 74 | | 53 | | 13 | | 2 | | 0.60984 | 1.081 | | 0.801-1.461 | |  | 188 | | 151 | | 40 | | 87 | | 67 | | 11 | 2 | 0.24343 | 1.187 | 0.890-1.583 |
| rs13333054 | IRF8 | G/A | 220 | 164 | | 20 | | 84 | | 48 | | 8 | | 2 | | 0.42371 | 1.14 | | 0.827-1.571 | |  | 210 | | 149 | | 19 | | 94 | | 63 | | 9 | 2 | 0.90520 | 1.018 | 0.754-1.375 |
| rs7238078 | MALT1 | C/A | 24 | 137 | | 240 | | 6 | | 47 | | 86 | | 1 | | 0.5263 | 1.113 | | 0.799-1.550 | |  | 21 | | 135 | | 220 | | 9 | | 49 | | 106 | 1 | 0.26100 | 1.199 | 0.873-1.646 |
| rs2283792 | MAPK1 | C/A | 130 | 197 | | 78 | | 37 | | 69 | | 34 | | 1 | | 0.12093 | 1.24 | | 0.945-1.629 | |  | 119 | | 185 | | 75 | | 48 | | 81 | | 37 | 1 | 0.44664 | 1.106 | 0.854-1.433 |
| rs17174870 | MERTK | G/A | 257 | 125 | | 22 | | 92 | | 41 | | 6 | | 2 | | 0.50916 | 1.123 | | 0.796-1.584 | |  | 242 | | 121 | | 15 | | 107 | | 45 | | 13 | 1 | 0.56232 | 1.098 | 0.800-1.509 |
| rs4648356 | MMEL1 (TNFRSF14) | A/C | 33 | 172 | | 197 | | 9 | | 66 | | 64 | | 2 | | 0.84698 | 1.03 | | 0.765-1.386 | |  | 30 | | 152 | | 195 | | 12 | | 86 | | 66 | 2 | 0.07310 | 1.29 | 0.976-1.705 |
| rs874628 | **MPV17L2(IL12RB1)^a)^** | A/G | 236 | 148 | | 20 | | 67 | | 64 | | 9 | | 1 | | **0.04452^a)^** | 1.366 | | 1.007-1.852 | |  | 208 | | 148 | | 22 | | 95 | | 64 | | 7 | 2 | 0.50343 | 1.109 | 0.820-1.499 |
| rs11154801 | MYB(AHII) | A/C | 61 | 195 | | 149 | | 22 | | 78 | | 40 | | 2 | | 0.19204 | 1.201 | | 0.912-1.581 | |  | 58 | | 186 | | 135 | | 25 | | 87 | | 54 | 2 | 0.65923 | 1.061 | 0.816-1.379 |
| rs4410871 | MYC | G/A | 227 | 154 | | 24 | | 74 | | 57 | | 9 | | 1 | | 0.54047 | 1.101 | | 0.809-1.499 | |  | 203 | | 151 | | 25 | | 98 | | 60 | | 8 | 2 | 0.20575 | 1.216 | 0.898-1.645 |
| rs228614 | NFKB1(MANBA) | A/G | 79 | 215 | | 111 | | 36 | | 70 | | 34 | | 2 | | 0.17771 | 1.206 | | 0.918-1.582 | |  | 81 | | 202 | | 96 | | 34 | | 83 | | 49 | 1 | 0.43963 | 1.107 | 0.855-1.434 |
| rs669607 | no gene | A/C | 84 | 222 | | 99 | | 32 | | 74 | | 34 | | 2 | | 0.74265 | 1.047 | | 0.797-1.373 | |  | 82 | | 205 | | 92 | | 34 | | 91 | | 41 | 1 | 0.81038 | 1.032 | 0.797-1.336 |
| rs12466022 | no gene | A/C | 30 | 143 | | 231 | | 13 | | 52 | | 75 | | 2 | | 0.36783 | 1.151 | | 0.848-1.563 | |  | 29 | | 136 | | 213 | | 14 | | 59 | | 93 | 2 | 0.85042 | 1.029 | 0.767-1.380 |
| rs13192841 | no gene | A/G | 43 | 181 | | 180 | | 10 | | 59 | | 71 | | 1 | | 0.13474 | 1.256 | | 0.931-1.693 | |  | 38 | | 163 | | 178 | | 15 | | 77 | | 73 | 2 | 0.77102 | 1.042 | 0.790-1.374] |
| rs7595037 | PLEK | G/A | 76 | 199 | | 128 | | 26 | | 68 | | 46 | | 1 | | 0.84064 | 1.029 | | 0.782-1.354 | |  | 67 | | 194 | | 117 | | 35 | | 73 | | 57 | 1 | 0.98709 | 1.002 | 0.772-1.301 |
| rs4613763 | PTGER4 | G/A | 9 | 83 | | 313 | | 1 | | 38 | | 101 | | 2 | | 0.43491 | 1.17 | | 0.789-1.736 | |  | 6 | | 84 | | 289 | | 4 | | 37 | | 125 | 2 | 0.68721 | 1.081 | 0.739-1.581 |
| rs2019960 | PVT1 | G/A | 22 | 140 | | 242 | | 6 | | 46 | | 88 | | 1 | | 0.47548 | 1.129 | | 0.809-1.574 | |  | 23 | | 122 | | 233 | | 5 | | 64 | | 97 | 2 | 0.98050 | 1.004 | 0.736-1.369 |
| rs1323292 | RGS1 | A/G | 274 | 114 | | 15 | | 91 | | 48 | | 0 | | 2 | | 0.82129 | 1.042 | | 0.728-1.493 | |  | 254 | | 113 | | 11 | | 111 | | 49 | | 4 | 2 | 0.84946 | 1.034 | 0.735-1.453 |
| rs180515 | RPS6KB1 | A/G | 158 | 184 | | 62 | | 49 | | 68 | | 22 | | 1 | | 0.52183 | 1.095 | | 0.829-1.447 | |  | 147 | | 170 | | 61 | | 60 | | 82 | | 23 | 1 | 0.95941 | 1.007 | 0.772-1.313 |
| rs140522 | SCO2 | A/G | 56 | 184 | | 164 | | 15 | | 61 | | 64 | | 1 | | 0.21308 | 1.201 | | 0.900-1.602 | |  | 50 | | 169 | | 160 | | 21 | | 76 | | 68 | 2 | 0.93199 | 1.012 | 0.773-1.325 |
| rs2744148 | SOX8 | A/G | 272 | 117 | | 15 | | 88 | | 48 | | 4 | | 1 | | 0.50360 | 1.124 | | 0.798-1.584 | |  | 246 | | 118 | | 14 | | 114 | | 47 | | 5 | 2 | 0.40333 | 1.155 | 0.824-1.618 |
| rs10201872 | **SP140^c)^** | G/A | 262 | 126 | | 17 | | 98 | | 40 | | 2 | | 2 | | 0.13526 | 1.32 | | 0.916-1.903 | |  | 265 | | 101 | | 13 | | 95 | | 65 | | 6 | 1 | **0.01214^c)^** | 1.5 | 1.091-2.063 |
| rs9891119 | **STAT3^b)^** | A/C | 155 | 193 | | 53 | | 44 | | 79 | | 15 | | 1 | | 0.51361 | 1.098 | | 0.829-1.454 | |  | 150 | | 185 | | 39 | | 49 | | 87 | | 29 | 1 | **0.00616^b)^** | 1.445 | 1.110-1.882 |
| rs1738074 | TAGAP | G/A | 173 | 169 | | 61 | | 64 | | 57 | | 18 | | 2 | | 0.42552 | 1.124 | | 0.843-1.499 | |  | 162 | | 163 | | 51 | | 75 | | 63 | | 28 | 1 | 0.84801 | 1.027 | 0.784-1.344 |
| rs802734 | THEMIS | A/G | 203 | 165 | | 35 | | 80 | | 49 | | 9 | | 2 | | 0.11870 | 1.284 | | 0.938-1.758 | |  | 200 | | 146 | | 29 | | 83 | | 68 | | 15 | 1 | 0.43307 | 1.121 | 0.843-1.491 |
| rs2293370 | TMEM39A(CD80) | A/G | 14 | 87 | | 303 | | 4 | | 38 | | 98 | | 2 | | 0.37251 | 1.185 | | 0.816-1.720 | |  | 13 | | 89 | | 277 | | 5 | | 36 | | 124 | 1 | 0.59879 | 1.104 | 0.763-1.598 |
| rs1800693 | TNFRSF1A | G/A | 100 | 178 | | 123 | | 28 | | 63 | | 49 | | 1 | | 0.18054 | 1.206 | | 0.917-1.587 | |  | 89 | | 157 | | 129 | | 39 | | 84 | | 43 | 2 | 0.20884 | 1.181 | 0.911-1.529 |
| rs6062314 | TNFRSF6B | G/A | 3 | 61 | | 341 | | 0 | | 21 | | 119 | | 1 | | 0.68286 | 1.112 | | 0.668-1.852 | |  | 3 | | 53 | | 323 | | 0 | | 29 | | 137 | 2 | 0.5957 | 1.134 | 0.713-1.804 |
| rs1077667 | TNFSF14 | G/A | 273 | 113 | | 17 | | 91 | | 40 | | 7 | | 1 | | 0.62467 | 1.09 | | 0.771-1.543 | |  | 257 | | 100 | | 21 | | 107 | | 53 | | 3 | 2 | 0.79040 | 1.047 | 0.748-1.464 |
| rs8112449 | **TYK2(ICAM3)^b)^** | A/G | 47 | 176 | | 181 | | 7 | | 59 | | 73 | | 1 | | **0.02681^b)^** | 1.409 | | 1.039-1.911 | |  | 43 | | 160 | | 175 | | 11 | | 75 | | 79 | 1 | 0.30504 | 1.159 | 0.874-1.535 |
| rs11581062 | VCAM1 | A/G | 205 | 165 | | 35 | | 69 | | 62 | | 9 | | 2 | | 0.88842 | 1.022 | | 0.757-1.380 | |  | 198 | | 151 | | 30 | | 76 | | 76 | | 14 | 1 | 0.2422 | 1.183 | 0.893-1.566 |
| rs4902647 | **ZFP36L1^d)^** | G/A | 135 | 190 | | 80 | | 39 | | 61 | | 40 | | 1 | | **0.03825^d)^** | 1.333 | | 1.015-1.751 | |  | 122 | | 175 | | 82 | | 52 | | 76 | | 38 | 1 | 0.74611 | 1.044 | 0.806-1.352 |
| rs1250550 | ZMIZ1 | A/C | 12 | 184 | | 67 | | 22 | | 174 | | 62 | | 2 | | 0.37465 | 1.118 | | 0.874-1.432 | |  | 37 | | 170 | | 168 | | 16 | | 81 | | 68 | 2 | 0.58232 | 1.08 | 0.821-1.420 |
| rs354033 | ZNF746 | G/A | 219 | 162 | | 23 | | 79 | | 49 | | 12 | | 1 | | 0.91372 | 1.017 | | 0.746-1.387 | |  | 197 | | 155 | | 26 | | 101 | | 56 | | 9 | 2 | 0.07724 | 1.315 | 0.970-1.781 |

**Table E in S1. p-value and ORs for allele correlation with clinical parameters.** column 1 = SNP with corresponding dbSNP rs-number; column 2 = gene name used by IMSGC & WTCCC2; column 3 = alleles present at the SNP locus (underlined are the risk alleles as identified by IMSGC & WTCCC2); column 4 to 6 = genotype distribution in Patients showing the clinical feature; column 7 to 9 = genotype distribution in Patients not showing the clinical feature; column 10 = identified risk allel with regard to the clinical feature; column 11 = one tailed allelic p-values calculated with one degree of freedom (df=1); column 12 = the odds ratios (OR) for the corresponding risk alleles; column 13 = 95% confidence interval (C.I.) of the corresponding OR; columns 14 to 23 = analogues to columns 4 to 13. Bold = p-valus < 0.05 before correction for multiple testing; ^a)^ = corresponds to risk allele in present study and GWAS; ^b)^ = inverse correlation of the risk allele from present study and GWAS; ^c)^ = corresponds to risk allele in present study but not to GWAS; ^d)^ = corresponds to risk allele from GWAS but not present study; ^e)^ = genotype distribution not in HWE; n_max_= maximum sample number available per clinical parameter.

|  |  |  |  |  |  |  | Ataxia in patients | | | | |  | Fam. MS in patients | | | | |
| --- | --- | --- | --- | --- | --- | --- | --- | --- | --- | --- | --- | --- | --- | --- | --- | --- | --- |
| SNP1 | SNP2 | Gene 1 | Gene 2 | SNP1 | SNP2 | OR for Pat vs. Contr | p val | OR | 95% C.I. | present | absent |  | p val | OR | 95% C.I. | present | absent |
| rs1520333 | rs949143 | IL7 | ARL6IP4 | AG | AG | 1.748 | 0.208 | 0.732 | 0.444-1.212 | 70/333 | 31/108 |  | 0.555 | 1.303 | 0.588-2.839 | 11/37 | 60/263 |
| rs669607 | rs12368653 | no gene | CYP27B1 | AA | GG | 0.481 | 0.188 | 0.45 | 0.130-1.395 | 380/25 | 135/4 |  | 1.000 | 0.965 | 0.197-6.404 | 46/2 | 310/13 |
| **rs10466829** | **rs13333054** | **CLECL1** | **IRF8** | GG | GG | 0.578 | 0.748 | 0.866 | 0.427-1.730 | 361/43 | 126/13 |  | **0.005** | 0.31 | 0.134-0.726 | 36/11 | 296/28 |
| rs10466829 | rs2546890 | CLECL1 | IL12B | GG | GG | 0.428 | 0.772 | 0.663 | 0.148-2.538 | 391/13 | 136/3 |  | 1.000 | 1.347 | 0.168-29.034 | 47/1 | 314/9 |
| rs7595037 | rs669607 | PLEK | no gene rs669607 | GG | AA | 0.454 | 0.428 | 0.54 | 0.123-2.008 | 389/16 | 135/3 |  | 0.624 | 0.584 | 0.110-4.117 | 46/2 | 315/8 |
| rs140522 | rs13333054 | SCO2 | IRF8 | AG | AG | 1.697 | 0.249 | 1.385 | 0.788-2.453 | 77/328 | 20/118 |  | 0.117 | 0.457 | 0.152-1.267 | 5/42 | 67/257 |
| rs13192841 | rs11154801 | no gene | MYB(AHI1) | AG | AA | 2.381 | 0.059 | 2.456 | 0.893-7.305 | 34/371 | 5/134 |  | 0.401 | 1.453 | 0.459-4.301 | 43/5 | 300/24 |
| rs669607 | rs2744148 | no gene | SOX8 | CC | AG | 2.040 | 0.148 | 0.614 | 0.304-1.250 | 28/377 | 15/124 |  | 1.000 | 0.997 | 0.281-3.191 | 44/4 | 296/27 |
| **rs140522** | **rs11154801** | **SCO2** | **MYB(AHI1)** | AG | AA | 2.178 | **0.024** | 3.59 | 1.026-15.010 | 30/376 | 3/135 |  | 0.753 | 0.661 | 0.103-3.076 | 2/46 | 20/304 |
| rs7200786 | rs9282641 | CLEC16A(CIITA) | CD86 | AA | AG | 2.948 | 0.157 | 2.597 | 0.724-11.079 | 22/384 | 3/136 |  | 0.446 | 1.595 | 0.346-6.326 | 3/45 | 13/311 |
| rs10201872 | rs7200786 | SP140 | CLEC16A(CIITA) | *GG* | AA | 1.601 | 0.171 | 1.466 | 0.845-2.560 | 84/322 | 21/118 |  | 0.852 | 1.079 | 0.490-2.333 | 11/37 | 70/254 |
| rs11154801 | rs2300603 | MYB(AHI1) | BATF | CC | GG | 0.340 | 0.465 | 3.114 | 0.400-66.232 | 9/396 | 1/137 |  | 1.000 | 1.351 | 0.169-29.127 | 47/1 | 313/9 |
| rs10466829 | rs7200786 | CLECL1 | CLEC16A(CIITA) | GG | GG | 0.506 | 0.662 | 1.176 | 0.463-2.898 | 385/20 | 131/8 |  | 0.334 | 3.257 | 0.448-66.546 | 47/1 | 303/21 |
| rs180515 | rs228614 | RPS6KB1 | NFKB1(MANBA) | GG | GG | 2.523 | 0.664 | 1.338 | 0.503-3.754 | 23/381 | 6/133 |  | 1.000 | 1.070 | 0.241-4.040 | 3/45 | 19/305 |
| rs7522462 | rs13192841 | C1orf106(KIF21B) | no gene | GG | AA | 2.458 | 1.000 | 1.089 | 0.454-2.695 | 25/376 | 8/131 |  | 0.753 | 0.595 | 0.093-2.746 | 2/46 | 22/301 |
| rs11129295 | rs12368653 | EOMES | CYP27B1 | GG | GG | 0.589 | 0.356 | 1.382 | 0.655-2.878 | 372/28 | 125/13 |  | 1.000 | 1.216 | 0.330-5.298 | 45/3 | 296/24 |
| rs10466829 | rs10201872 | CLECL1 | SP140 | GG | *AG* | 0.566 | 0.823 | 0.825 | 0.291-2.217 | 384/21 | 133/6 |  | 1.000 | 1.117 | 0.233-7.315 | 46/2 | 309/15 |
|  |  |  |  |  |  |  |  |  |  |  |  |  |  |  |  |  |  |
|  |  |  |  |  |  |  | Paresis in oatients | | | | |  | Impaired vision in patients | | | | |
| SNP1 | SNP2 | Gene 1 | Gene 2 | SNP1 | SNP2 | OR for Pat vs. Contr | p val | OR | 95% C.I. | present | absent |  | p val | OR | 95% C.I. | present | absent |
| rs1520333 | rs949143 | IL7 | ARL6IP4 | AG | AG | 1.748 | 0.617 | 0.888 | 0.532-1.486 | 73/329 | 28/112 |  | 1.000 | 0.996 | 0.608-1.637 | 70/306 | 31/135 |
| rs669607 | rs12368653 | no gene | CYP27B1 | AA | GG | 0.481 | 1.000 | 0.905 | 0.343-2.295 | 381/22 | 134/7 |  | 1.000 | 1.008 | 0.415-2.393 | 356/20 | 159/9 |
| rs10466829 | rs13333054 | CLECL1 | IRF8 | GG | GG | 0.578 | 0.520 | 0.757 | 0.366-1.538 | 358/44 | 129/12 |  | 0.544 | 0.806 | 0.413-1.558 | 335/41 | 152/15 |
| rs10466829 | rs2546890 | CLECL1 | IL12B | GG | GG | 0.428 | 0.261 | 0.399 | 0.062-1.868 | 388/14 | 139/2 |  | 0.586 | 1.364 | 0.433-4.159 | 366/10 | 161/6 |
| rs7595037 | rs669607 | PLEK | no gene rs669607 | GG | AA | 0.454 | 0.792 | 0.753 | 0.207-2.477 | 387/15 | 137/4 |  | 0.453 | 0.591 | 0.163-1.937 | 361/15 | 163/4 |
| rs140522 | rs13333054 | SCO2 | IRF8 | AG | AG | 1.697 | 0.446 | 1.241 | 0.718-2.159 | 75/327 | 22/119 |  | 0.545 | 1.186 | 0.710-1.989 | 70/306 | 27/140 |
| rs13192841 | rs11154801 | no gene | MYB(AHI1) | AG | AA | 2.381 | 0.850 | 1.180 | 0.520-2.752 | 30/373 | 9/132 |  | 0.857 | 1.138 | 0.527-2.500 | 28/349 | 11/156 |
| rs669607 | rs2744148 | no gene | SOX8 | CC | AG | 2.040 | 0.720 | 0.896 | 0.427-1.908 | 31/372 | 12/129 |  | 1.000 | 1.034 | 0.503-2.154 | 30/346 | 13/155 |
| rs140522 | rs11154801 | SCO2 | MYB(AHI1) | AG | AA | 2.178 | 0.412 | 1.616 | 0.618-4.466 | 27/376 | 6/135 |  | 0.702 | 0.879 | 0.395-1.985 | 22/355 | 11/156 |
| **rs7200786** | **rs9282641** | **CLEC16A(CIITA)** | **CD86** | AA | AG | 2.948 | **0.036** | 4.196 | 0.942-26.097 | 23/381 | 2/138 |  | 0.274 | 1.826 | 0.633-5.659 | 20/357 | 5/163 |
| **rs10201872** | **rs7200786** | **SP140** | **CLEC16A(CIITA)** | *GG* | AA | 1.601 | 0.71 | 0.895 | 0.541-1.486 | 76/328 | 29/112 |  | **0.007** | 2.007 | 1.164-3.486 | 84/293 | 21/147 |
| rs11154801 | rs2300603 | MYB(AHI1) | BATF | CC | GG | 0.340 | 1.000 | 0.716 | 0.104-3.687 | 395/8 | 138/2 |  | 0.297 | 0.246 | 0.012-1.907 | 367/9 | 166/1 |
| rs10466829 | rs7200786 | CLECL1 | CLEC16A(CIITA) | GG | GG | 0.506 | 0.506 | 1.378 | 0.562-3.308 | 384/19 | 132/9 |  | 0.836 | 1.073 | 0.439-2.568 | 358/19 | 158/9 |
| rs180515 | rs228614 | RPS6KB1 | NFKB1(MANBA) | GG | GG | 2.523 | 0.383 | 1.710 | 0.603-5.220 | 24/379 | 5/135 |  | 1.000 | 0.986 | 0.415-2.397 | 20/356 | 9/158 |
| rs7522462 | rs13192841 | C1orf106(KIF21B) | no gene | GG | AA | 2.458 | 0.217 | 2.032 | 0.728-6.127 | 28/372 | 5/135 |  | 1.000 | 1.013 | 0.447-2.341 | 23/352 | 10/155 |
| rs11129295 | rs12368653 | EOMES | CYP27B1 | GG | GG | 0.589 | 0.854 | 1.057 | 0.483-2.274 | 369/30 | 128/11 |  | 0.602 | 0.801 | 0.367-1.717 | 341/30 | 156/11 |
| rs10466829 | rs10201872 | CLECL1 | SP140 | GG | *AG* | 0.566 | 1.000 | 1.000 | 0.375-2.568 | 383/20 | 134/7 |  | 0.054 | 2.189 | 0.943-5.074 | 363/14 | 154/13 |

**Table F in S1. p-value and ORs for genotype/genotype combinations according to Model A) correlated for clinical parameters.** column 1 = SNP1; column 2 = SNP2; column 3 and 4 = suggested genes by IMSGC and WTCCC2 for SNP1 and SNP2; column 5 and 6 = genotypes included in the genotype/genotype combinations – risk alleles for the corresponding genes are underlined (italic = deviation of previously identified risk alleles); column 7 = the odds ratios (OR) calculated when comparing patients vs. controls – see Supplementary Table 3; column 8 = p-value for the genotype/genotype combinations correlating with clinical parameters – in case the initial OR (column7) is <1, samples showing the genotype/genotype combinations were treated as not having the risk factor and the rest were treated as having the risk factor, and in case the initial OR (column7) is >1, samples showing the genotype/genotype combinations were treated as having the risk factor and the rest were treated as not having the risk factor; column 9= odds ratio (OR) for that given genotype/genotype combination; column 10 = 95% confidence interval (C.I.); column 11 = number of samples showing the clinical feature – left of the diagonal slash equals to the patients with the risk combination / right of the diagonal slash equals to the patients without the risk combination; column 12= analogous to column 11 only for samples not showing the clinical feature; column 13 to 17 = analogues to columns 8 to 12. bold = significant p-values before correction for multiple testing.
